# Modularity of food-sharing networks minimises the risk for individual and group starvation in hunter-gatherer societies

**DOI:** 10.1371/journal.pone.0272733

**Published:** 2023-05-10

**Authors:** Francisco Plana, Jorge Pérez, Andrés Abeliuk

**Affiliations:** 1 Department of Computer Science, Universidad de Chile, Santiago, Chile; 2 Millennium Institute Foundational Research on Data, Santiago, Chile; 3 National Center for Artificial Intelligence (CENIA), Santiago, Chile; Universidad de Burgos, SPAIN

## Abstract

It has been argued that hunter-gatherers’ food-sharing may have provided the basis for a whole range of social interactions, and hence its study may provide important insight into the evolutionary origin of human sociality. Motivated by this observation, we propose a simple network optimization model inspired by a food-sharing dynamic that can recover some empirical patterns found in social networks. We focus on two of the main food-sharing drivers discussed by the anthropological literature: the reduction of individual starvation risk and the care for the group welfare or egalitarian access to food shares, and show that networks optimizing both criteria may exhibit a community structure of highly-cohesive groups around special agents that we call hunters, those who inject food into the system. These communities appear under conditions of uncertainty and scarcity in the food supply, which suggests their adaptive value in this context. We have additionally obtained that optimal welfare networks resemble social networks found in lab experiments that promote more egalitarian income distribution, and also distinct distributions of reciprocity among hunters and non-hunters, which may be consistent with some empirical reports on how sharing is distributed in waves, first among hunters, and then hunters with their families. These model results are consistent with the view that social networks functionally adaptive for optimal resource use, may have created the environment in which prosocial behaviors evolved. Finally, our model also relies on an original formulation of starvation risk, and it may contribute to a formal framework to proceed in this discussion regarding the principles guiding food-sharing networks.

## Introduction

### Food-sharing and its relevance for the evolution of human cooperation

Human beings are social animals that have built many forms to organize their life in community due to their remarkable ability to coordinate and jointly work to attain common goals. A fingerprint of these behaviors can be tracked in the myriad types of social networks human beings develop, such as family, friends, and communities of interest. Namely, it has been argued that a distinctive attribute of social networks is that they have communities or modules, that is, cohesive groups of agents more densely connected among them than with the rest of the network. This feature may help to explain other special attributes of social networks [1].

One of the key factors for the successful adaptation of the human species to almost every habitat on the planet along its evolutionary history [2], is the unique complexity of its patterns of food sharing, which extends well beyond infancy lactation to the whole life and across adults and families [3]. These patterns may have emerged as a response to a greater offspring dependency associated with the development of a larger brain requiring a nutrient-rich diet, more difficult feeding strategies, and foraging skills that may not develop until late in life, in contrast to other wild primates having more predictable diets [3–5]. The large human brains may have had a central role both to the formation of modular social networks enabling adaptive collective computation, to the optimization of foraging behaviors [6]. These behaviors ensuring the most elemental subsistence may have given the material basis for inter-generational accumulation of cultural innovations [7], and the whole range of social interactions carried out by hunter-gatherer bands, such as cooperative breeding [8, 9], establishing political alliances and reinforcing social bonds [10–13], labor sharing [14, 15] and costly signaling or show-off for mating purposes [16, 17]. Therefore, investigating the evolutionary formation of food-sharing networks may yield important insights for the study of social dynamics. With this in mind, we aim to theoretically contrast the hypothesis that the modular character of food-sharing networks, may be an optimal response for avoiding the risk of individual and collective starvation.

### Characteristics and drivers of food-sharing networks

It has been reported that empirical food-sharing networks present the same traits of other general social networks such as *degree assortativity* [18], or positive correlation on neighbors degree, *transitivity* or *global clustering* [19], the fact that agent *i* is likely connected to *k* if *i* is connected to *j* and *j* to *k*. These two network characteristics may be an indication of communities [1]. Other such characteristics are *reciprocity* [20], which is a significant proportion of agent pairs with bidirectional connections, and a *multilevel* structure [21] of cohesive groups hierarchically organized. Among the few theories aiming to explain some of these network patterns, it has been argued that the multilevel structure would be related to the efficiency of information flow [22] which may in turn, facilitate the diffusion of cultural innovations [23]. We think that, though the efficiency information flow may be an important force shaping social networks, it does not fully serve as an explanation for the evolution of food-sharing networks. On the one hand, it does not have a clear connection to the usual motives for food-sharing discussed in the evolutionary anthropology literature [3]. On the other hand, the theory of cultural innovation may impose high cognitive requirements [24] which do not explain, for example, why these multilevel networks may be observed also in other social animals and non-mammals [25]. Another work [21], though it is not a theory of multilevel structure, gives suggestive evidence relating three nested network levels, the household, the cluster, and the camp, to distinct social and economic cooperative functions: intersexual provisioning of a couple and their dependent children in a household, kin provisioning, and risk reduction reciprocity, respectively.

The motives for food-sharing most supported by empirical field studies are kinship, need, reciprocity, and inter-household distance, where the latter is usually considered a proxy of the tolerated theft motive [20, 26–32]. The costly signaling hypothesis, which claims that food-sharing is made in order to gain status, has received minor support in recent works as a factor for food transfers, though status is an important driver of reproductive fitness [33–35]. Probably the most accepted causal interplay between these motives is the following. Reciprocal altruism, or contingency on past transfers, arises as an important, or the strongest, predictor in most studies, usually explained as a means of lowering the uncertainty of food generation [10, 20, 21, 26–32]. Kinship, or the preference for relatives, is another important driver of food transfers [10, 20, 21, 26, 27, 29, 31, 32], which appears to be as a bias, or partner-selection mechanism, interacting with reciprocity or need by increasing the associations to transfers with respect to non-kin [20, 26, 27, 31]. Tolerated theft, or sharing when the benefits of hoarding are outweighed by the cost of retaining the resource, is often a less important but significant motive of transfers [29, 36], though it may be empirically difficult to disentangle from the alternative explanations [10, 29, 36]. Some works have suggested that tolerated theft may be an evolutionary precursor of reciprocal altruism, given that the former is more common among other animals, and the latter has higher cognitive requirements that makes it almost nonexistent in non-human animals [4, 37]. Finally, the motive of need, or giving to those with a lower relative net energy production, often reflected in a positive correlation of transfers with receiving household size, is also an important force guiding food transfers [20, 21, 27, 31, 32].

### The issue with need motive and a possible solution

The motive of need for food transfers has a difference with the other reviewed motives: it is not evolutionary adaptative, at least in the usual individual fitness models [38]. These models assert that cooperation requires to be assortative to be stable, that is, directed to other cooperative individuals [39]. This prediction has only partial support in real-world food sharing, where it is common that families or foragers in relative need like the elderly, tend to receive more resources [21, 31, 32] or that free-riders, those who take shares, but do not reciprocate, are not excluded from sharing [40]. A possible theoretical remedy that has been suggested [32] is to consider a diminishing returns value function of the transfers, which may produce a long-term equilibrium in transfers. In a previous publication [41], it was argued that assuming reciprocal altruism, sharing is an optimal individual strategy for values following a diminishing returns function of the shares, rather than just the raw quantity of food.

We propose a distinct intuition guiding the abstraction of our model. We propose that the argument for using diminishing returns comes from the fact that the essential problem a hunter-gatherer community is solving when sharing food, is to satisfy the need given by hunger. This is the intuition of the law of diminishing marginal utility given by Mill [42], stating that if there is enough of a good to satisfy a basic level of need, then each additional increment of the good satisfies the need less than the previous increment. The basic social dilemma for the food-sharing network then comes from the conflict between the goal of satisfying the own need and the need of other people. If the satisfaction of the hunger need is defined as the goal of minimizing the risk of starvation, or the time without eating, then the own need may be embedded in the tolerated theft or reciprocal altruism motives, while the hunger of other people may be represented by the need motive. We do not explicitly incorporate kinship in our model in order to keep it simple. It has been suggested that many kinship configurations may be a product of sharing patterns [12, 43, 44], which suggests that the basic social dilemma posed is enough to recover the basic dynamics of food-sharing networks, such as cohesive groups given by nuclear families with a few interconnections that comprise a cluster of akin families [21].

### Our proposed model for food-sharing networks, its assumptions, and contributions

In this work, we propose a normative model [45] for food-sharing networks, formulated as a network optimization. That is, the model does not aim at fitting real networks, but rather derives idealized results from a set of simplified assumptions. The optimizing goals correspond to the minimization of starvation risk, either at the individual level or as a group. These goals are named in the model as *Reduction of Variability* (RV) and *Welfare* (WEF). The assumptions taken by the model are the following.

- An individual who has food eats only the amount required to survive, and shares all the remaining food. This is inspired by the empirical finding that giving a high proportion of food is a common practice [41].
- The structural imbalance between production and consumption leading to adult sharing to provide infants and juveniles [3], is implemented by a special set of network nodes called *hunters*, which are those who inject food into the network. The number of hunters in a network of *N* agents corresponds to the parameter *nh*.
- Since large and unpredictable food items such as meat or honey are more widely shared [4, 40, 41, 46], the model poses that at each time step, there is an amount *F* of food able to feed several individuals that is produced with certain probability *ph*. These quantities, *F* and *ph*, are parameters of the model.
- Any additional motives for sharing, like kinship, which would introduce, for example, preferential sharing partners, are abstracted. Therefore, given a food-sharing network, and an agent that receives food, this agent chooses randomly one of their neighbors to share. Thus, the transfers of food in the model correspond to recurring exchanges among stable connections, that may well reflect the food sharing in a small community formed by a few akin families. This assumption is also consistent to the choice taken for sampling the population size parameter used in the simulation. Technologies for storing food are not considered in this model. These assumptions are taken to keep the model simple.

The model aims to recover the constraints for optimal network organization derived from material transfers of food under the stated assumptions. This emphasis in the material dimension of food-sharing, does not look for neglecting that this behavior is a complex sociocultural phenomenon [47]. Rather, the model shows that evolutionary models of social dynamics may benefit from a resource distribution perspective and the explicit inclusion of the group level of analysis, given that the typical small-world structure of social networks given by the presence of communities, is recovered under the simultaneous optimization of the two goals, that is, the individual but also the group survival. Specifically, the contributions of our work are the following.

First, networks optimizing both criteria may exhibit a community structure of cohesive groups under stringent conditions of food supply. These networks are resemblant to the food-sharing networks observed by Dyble et al (2016) [21], where there are cohesive groups corresponding to households provisioned by an adult couple, and a set of households forms a cluster with a small number of inter-household connections. This model result suggests that this organization of nuclear families may have been evolutionarily functional for resource distribution optimality. We have additionally obtained that optimal welfare networks, where each hunter is connected to one big, homogeneous and dense group of non-hunters, resemble social networks that promote more egalitarian income distribution in a lab gift game reported by Chiang (2015) [48]. This model result is consistent with the view that social networks structured originally according to ecological considerations, may have created the environment in which prosocial tendencies and equity response elicitation evolved [49, 50]. Finally, distinct distributions of reciprocity are obtained for each optimization regime, which may be consistent with the empirical finding [51] that often the sharing occurs in waves: first among hunters, and then hunters with their families. This model result gives a broader picture of the usual notion that reciprocity is driven by the minimization of food production uncertainty [28].

### Paper structure

The rest of the paper is structured as follows. In the next section, “Materials and methods”, all the modeling assumptions and analysis techniques are described at a high level. Several details are set aside for the “Supporting information” section at the end of the article. After the methods section, there is a section of results where a description is given regarding the structural behaviors observed in the three types of optimal networks analyzed, WEF, RV, and simultaneous optimization of both criteria. The interpretation of these results is addressed next in the Discussion Section. We end the article with the conclusions.

## Materials and methods

This section aims to briefly explain the modeling, implementation and analysis approach adopted in this work. Several details are left out to be consulted in the Appendix of S1 File. The next sections go as follows. In the first two sections we describe the abstract protocol of foodsharing among agents, and the consequent model of probabilities of receiving food. Next section describes how the optimizing goals, welfare, and reduction of risk, are designed as a function of these probabilities. Next, we show the solution concept of the optimization model, and how this model is implemented by evolutionary algorithms. The section is finalized by the pipeline of analysis, which describes how the output of the optimization model is processed using the following data analysis techniques: description by network features, clustering, and classification trees.

### Food-sharing protocol and assumptions

We propose a network optimization model in which each agent is a node in a loop-free directed network *D* = (*V*, *E*). There is a special set *H* ⊆ *V* of agents in *D* that we call *hunters*. Agents in *H* are, intuitively, responsible for injecting *food* in the network by *hunting preys*. We assume that there is a real number *ph* ∈ [*0*,*1*], a parameter of the model, that determines the probability that an agent in *H* hunts at each time step. The prey obtained in every hunt is enough to feed *F* agents (including the hunter). The value *F* is a fixed positive integer that is also a parameter of the model. The food resulting from a hunt is *shared* from the hunters to the rest of the network, only through network edges *e* ∈ *E*, and is used to feed the agents as we next explain. These assumptions implement the following two empirical facts: food items more widely shared are those unpredictable and coming in large packages [4, 40, 41, 46], and the fact that all agents require eating for survival, but only some of them can produce food [3].

We assume that all the exchanges of food derived from one successful hunt, occur at the same time step where the prey is caught. When an agent *v* ∈ *V* receives *f* units of food from a neighbor, it consumes a single unit of food. After this, agent *v* chooses uniformly at random one of its neighbors, say *v*′, and sends all the remaining food (*f* − 1 units) to *v*′. This assumption is inspired by the empirical finding that giving a high proportion of food is a common practice [41]. Notice that this sharing from *v* to *v*′ is possible only if *v* initially received *f* > 1 units of food. Additional food received in the same time step, cannot be mixed with other food packages, nor stored for later usage. For simplicity, we assume that a hunt by a hunter is equivalent to receiving *F* units of food, though in this case the food is not received from a neighbor but from the environment. Thus, after feeding itself, a hunter sends *F* − 1 units of food to a random neighbor. These assumptions are taken to keep the model simple and to abstract the possible motives for agents’ actions.

In our model, time is discrete, and at each time step, every hunter may or may not hunt with the same probability *ph*. Due to previous assumptions, those agents at distance greater than (*F* − 1) to every hunter, simply cannot receive food. Thus, every node *v* ∈ *V* in the network at a distance at most (*F* − 1) to some hunter, may or may not eat depending on whether *v* receives food, either by hunting or by sharing. In order to model the effect of feeding an agent, we assume that agents have a *life span* of n∈N, and that there is a *critical time window* *k* < *n*, in which, a run of *k* consecutive time steps without receiving food turns out to be seriously detrimental for every agent, resulting in its death. Only non-dead agents can hunt, eat, send or receive food. Therefore, the agents will try to eat as frequently as possible, to avoid spending too many rounds without eating.

To sum up, the parameters of our model are the probability of hunting in a time step (*ph*), the units of food produced by a prey (*F*), the number of time steps of life span (*n*), and the maximum number of time steps an agent can survive without eating (*k*). Moreover, a network in our model is defined by *D* = (*V*, *E*) plus a set *H* ⊆ *V* of hunters. We note that, even when fixing the parameters of the model, different networks behave in distinct ways depending on their connectivity (and the presence of hunters).

### Probability of eating

**Fig 1:**
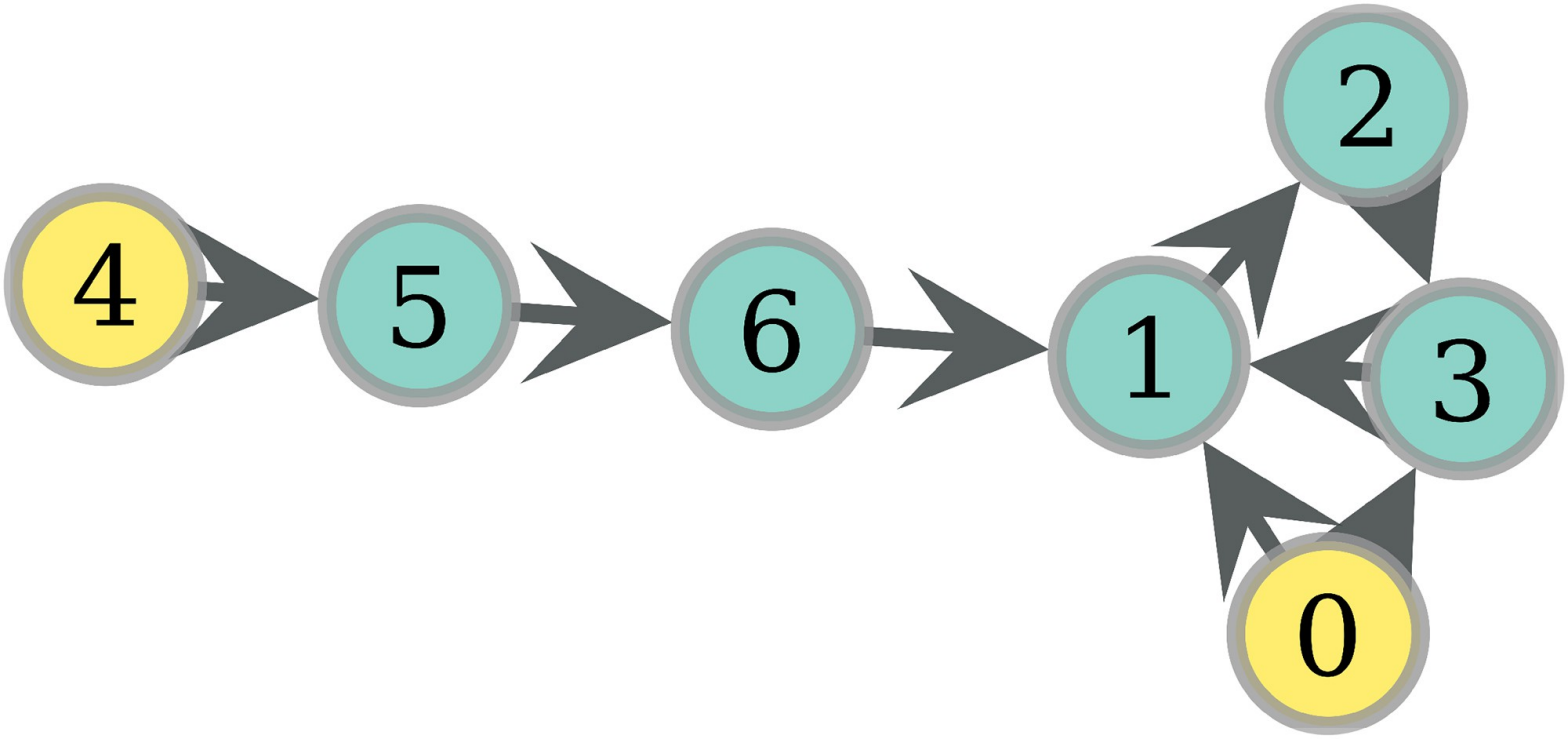
A network with 7 nodes and 2 (yellow) hunters.

In this section, we give some intuition about how the probabilities of eating behave according to the previously described protocol of food sharing. The interested reader may look at S1 Appendix of S1 File in order to see the analytical derivation of these probabilities. For now, let us see an example. Fig 1 shows a network with 2 hunters, 0 and 4. Assume that *ph* = 0.2 and obtain the probabilities of eating according to (4) (S1 Appendix of S1 File) for distinct values of *F*, displayed in Table 1. For *F* = 2, 5 is surely fed by 4 with the probability of hunting *ph*, while 1 and 3 get a distributed chance of receiving food. In the case of *F* = 3, now 6 is also fed with probability 0.2, and there are two 2–walks from 0: {(0, 1), (1, 2)} and {(0, 3), (3, 1)}. Since 1 is fed by the 2 equiprobable walks, its probability of eating is 2 * 0.1. When *F* = 4, 1 gets food by another walk, raising this probability to 0.36 = 0.2 + 0.2 − (0.2)^2^ thanks to Inclusion-Exclusion. Finally, notice in *F* = 5 that, though node 3 is reached twice by one walk, this does not increase the probability of eating since this probability does not measure the quantity of food, but rather the distinct events of food supply.

**Table 1:** Probabilities of eating, distinct F values.

*pe*(·)	0	1	2	3	4	5	6
*F*							
2	0.2	0.1	0	0.1	0.2	0.2	0
3	0.2	0.2	0.1	0.1	0.2	0.2	0.2
4	0.2	0.36	0.2	0.2	0.2	0.2	0.2
5	0.2	0.36	0.36	0.2	0.2	0.2	0.2

### Optimizing criteria

We have said our model may have two possible goals, welfare, the egalitarian group access to food, and avoidance of individual starvation. Since our model implements food exchanges as a probabilistic protocol, the access to food resources is expressed as the probability of eating. Our model is framed as the minimization of an objective function, thus, we aim to penalize the dispersion of this probability among agents. Next, we formalize the two goals as minimization objectives.

**Definition 0.1** (Welfare). Given fixed values for variables *ph*, *F*, *n*, *k* and *nh* = |*H*|, *Welfare* (WEF) criterion for minimization is the standard deviation of probabilities of eating over network nodes.

Now, the minimization of individual starvation risk is formalized as follows. We can think of the time steps in our model, and the possibility of eating at each time as a sequence of Bernouilli trials, where a success is given by the event of not receiving food, otherwise we have a failure. That is, at each time step, node *v* obtains a success with probability 1 − *pe*(*v*), where *pe*(*v*) is computed as explained in the previous sections. Since network nodes attempt to avoid *k* consecutive steps without food, or a success run of length *k*, in order to stay alive, a possible formulation of reduction of starvation risk can be expressed as the minimization of the number *G*_*n*,*k*_ of success runs of length greater than or equal to *k* in *n* trials. The distribution and moments of this random variable can be computed exactly with a Markov chain embedding technique [52], which allows defining this criterion as the minimization of the sum of the expected value and standard deviation of *G*_*n*,*k*_.

The random variable *G*_*n*,*k*_ defined above is expressed as an integer number of runs. One can normalize this variable *G*_*n*,*k*_ by the total maximum number of runs of length *k* that may be observed in *n* trials. In this way, this criterion could be interpreted as the probability of dying within the life span -or observing a *k*–length (or greater) run with no food in *n* trials- as a function of the probability of receiving food at each trial. Since the number of runs of length at least *k* within *n* trials is maximized when all the runs have the minimum length *k*, the normalizing denominator may be estimated as the floor of $\frac{n+1}{k+1}$, where the “+1” appears to count the spaces necessary to split a new run from the previous one. With these ingredients, we formally define the second minimization criterion.

**Definition 0.2** (Reduction of variability function). Given fixed values for variables *n*, *k*, and 1 − *pe*(*v*) for the probability of a success, let *G*_*n*,*k*_ be the random variable given by the number of success runs of length greater than or equal to *k* in *n* trials. We will refer to the expected value and the standard deviation of this variable as $E(G_{n,k}\left(pe\left(v\right)\right))$ and *σ*(*G*_*n*,*k*_(*pe*(*v*))). The *Reduction of Variability* of food intake (RV) function of the probability of eating, is defined as $RV\left(\cdot \right)=\frac{k+1}{n+1}\cdot \left(E\left(G_{n,k}\left(\cdot \right)\right)+\sigma \left(G_{n,k}\left(\cdot \right)\right)\right)$.

**Definition 0.3** (Individual starvation risk criterion). Given fixed values for variables *ph*, *F*, *n*, *k* and *nh* = |*H*|, the *Individual starvation risk* criterion for minimization is the average over network nodes of the RV function, evaluated at the respective probability of eating of each node. For brevity, and without causing ambiguity (since the function is for nodes, and the criterion for networks), we refer also to this criterion as RV.

**Fig 2:**
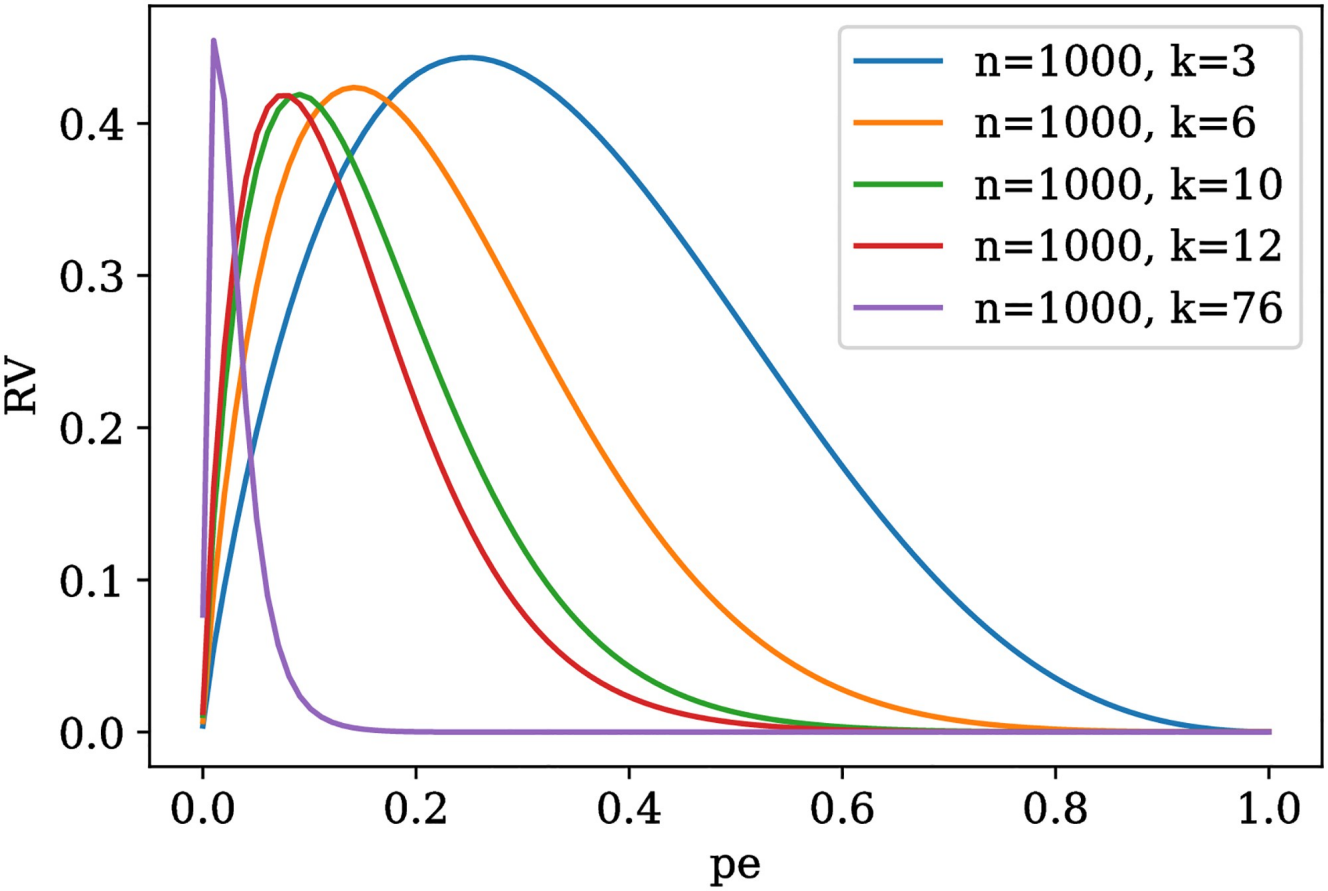
Reduction of variability function of the probability of eating. The X-axis is the probability of eating or receiving food, as defined in the last sections. The vertical axis is the Reduction of variability (RV) function of this probability. Each curve corresponds to a distinct instance of the variables *n*, the number of life span time steps, and *k*, the maximum number of time steps an agent can survive without eating.

We have taken the average to approximate the individual agent risk since this function is not a robust statistic of central tendency [53], and may be extremely affected by the presence of outliers. That is, the mean of RV over network nodes may be minimized by a few number of agents with low RV, while a great number still experiment high starvation risk. Now, in Fig 2 we can observe distinct instances of the RV function, for different values of *k* and *n* = 1000. These RV curves have a maximum value corresponding to some value *pe*· ∈ (*0*, *0.5*), and the larger the length *k* of the run, the smaller the probability of eating *pe*· at which this maximum is obtained, which makes intuitive sense. In the following, we will continue working with the values *k* = 10 and *n* = 1000, since from inspection of this plot, this function displays all relevant behaviors within a non-degenerate probability domain. Therefore, the resting free variables, with which we will work are *F*, *ph* and *nh* = |*H*|.

### Model solution

#### Definitions

We need to define the set of solutions for our minimization model. At first sight, this set corresponds to the directed graphs of *N* nodes, if we have a population of *N* agents. However, there is a huge number of non-isomorphic networks sharing the same optimizing costs due to subgraphs in the network that do not contribute to the optimizing criteria functions. We remedy this by defining the domain set of our optimization model as the quotient set of the set of networks of size *N*, with respect to an equivalence relation given by a suitable definition of isomorphism that accounts for this fact. This definition impacts the evolutionary algorithms used to compute model optima, which are described in the next section. The interested reader may review the isomorphism definition and examples in the S4 Appendix of S1 File.

Finally, we give the solution concepts used in our minimization problems. For single criterion minimization, a solution network is given by a *Local Minimum*, which is a network of minimal value with respect to every possible change of network arc. In the case of simultaneously minimizing both criteria, we use the standard notion of *Pareto Optimality* [54], defined next.

**Definition 0.4** (Dominance). A vector $\vec{u}=\left(u_{1},u_{2}\right)\in R^{2}$ is said to *dominate* vector $\vec{v}=\left(v_{1},v_{2}\right)\in R^{2}$, which is denoted by $\vec{u}⪯\vec{v}$, if ∀*i* ∈ {1, 2}, *u*_*i*_ ≤ *v*_*i*_, and ∃*i* ∈ {1, 2}, such that *u*_*i*_ < *v*_*i*_.

**Definition 0.5** (Pareto Optimality). Let *F*(⋅) = (*f*_1_(⋅), *f*_2_(⋅)) be a set of objective real-valued functions. A point *x* from the domain of *F* is said to be *Pareto Optimal*, if there is no *x*′ in the domain of *F*, such that *F*(*x*′) dominates *F*(*x*).

Defined in this way, a point *x*^⋆^ is Pareto optimal for a given objective function *F*(⋅) = (*f*_1_(⋅), *f*_2_(⋅)), if there is no feasible point *x*, such that *f*_1_(*x*) < *f*_1_(*x*^⋆^) and simultaneously, *f*_2_(*x*) < *f*_2_(*x*^⋆^). The set of all Pareto optima is the *Pareto optimal set*, and its image via function *F* is the *Pareto front*.

#### Model implementation

We have tackled our minimization problem with evolutionary algorithms, due to their versatility to accommodate the model assumptions, and their ability to manage several candidate solutions. We have used the known (*μ*, λ)–procedure, where *μ* is the population size, at each generation every individual gives birth to λ offspring by a feasible mutation, and from this set *μ* descendant are selected for the next generation population. This procedure is recommended as a way to preserve the diversity of solutions [55]. For single objective offspring selection, we have used *tournament selection* of size *t* = 12, that is, *t* individuals are randomly selected from the offspring population, and the one with the best function value is selected. This relatively high value of *t* is chosen to increase the likelihood of selecting local optima, something usually referred to as *exploitation* or *selective pressure*. In most cases, we have used *μ* = 1000 and λ/*μ* = 8 in order to increase the selective pressure. The multi-objective case employs the NSGA-IIR algorithm [56] as an offspring selection mechanism. This algorithm is an improved version of the well-known NSGA-II [57], that takes advantage of its relatively low computing and storage complexity, but that solves some instabilities that may appear when two or more solutions share the same objective values [56]. We have implemented these algorithms on the DEAP library [58] for Python, a package devised for the modular design of evolutionary algorithms. The networks are implemented with the module graph-tool [59], a Python library for manipulation and statistical analysis of graphs. We have taken a series of implementation decisions to obtain results more efficiently. The interested reader may review them in the S4 Appendix of S1 File.

### Pipeline of analysis

The outcome of the evolutionary algorithms described previously is a set of networks, for every sampled setting of parameters *F*, *ph*, and *nh* = |*H*|. In the S2 Appendix of S1 File, the structure of the simulation carried out is explained, which comprises the choice of sampled parameters. Now, to obtain a high-level description of the set of different optima networks, we processed the data according to the following steps. For each network, a set of features is computed to build a table of networks and features, for either single criteria or multiobjective case. See Tables 1, 2 in the S3 Appendix of S1 File to consult the sizes of these datasets. These networks are then clustered by their feature similarity, where each group depicts a set of qualitatively similar networks. Finally, a decision tree to discriminate distinct groups is computed, which permits an objective split of groups in terms of the feature values. Next, we review in more detail each of these steps.

#### Descriptive features

Here we explain the features used to describe the networks. These features correspond mostly to simple metrics of network topology that can be efficiently obtained. We next list them grouped by their reason to be included as potentially useful descriptors. An important remark is that, though these descriptors are used in their original scale to build the decision trees, they are standardized for the clustering stage since this is recommendable for these methods. We used the generalized spatial sign technique to accomplish this [60], with quadratic radial function and *k*–step least trimmed squares for the location estimator, since it delivers robustness to estimators based on co-variances in the presence of potential outliers.

- The minimization of WEF may reduce the dispersion in the number of walks from hunters to every node, since the probabilities of eating depend on them. We aim to detect this scenario with the proxy variables *standard deviation of out-degree and in-degree distributions*.
- To identify potential community or group structure, we have added several variables. A very simple measure of this is the *number of strongly connected components*. We added the mean of *local clustering* [61], which estimates the probability that two neighbors are connected, and the *in-degree and out-degree correlation or assortativity*, since they have been associated with the presence of dense groups [1]. Assortativity has also been associated to the presence of *reciprocity* [62], which we define as the proportion of connected vertex pairs having bidirectional arcs. This is a prevalent pattern found in social networks [63]. We have finally considered *network modularity* [64], an index of network partitioning into dense groups with few inter-group connections. We obtain candidate optimal partitions by running a Markov chain Monte Carlo method several times [65].
- The minimization of RV may produce important variations in the overall connectivity of the network, depending on the specific value of *ph*. We will measure network connectivity with the *mean of out-degree distribution*, and the *mean of in-degree of hunters*. The latter variable may also diminish in some WEF minima.
- The 2 network costs to be minimized, RV and WEF.

The assortativity indexes have been used only for WEF analysis, since the other type of optima sometimes presents indefinite values.

#### Feature-similarity-based clustering

The stage of clustering is performed by the successive application of tSNE and OPTICS algorithms. The first of them, the *t-Distributed Stochastic Neighbor Embedding* [66], was chosen by its formulation which finds a low-dimensional map preserving the local similarity structure of the data. This implements one of the goals of our analysis which is to group similar networks, where their similarity is expressed in terms of their feature values. Another important reason to choose this technique is its remarkable ability to produce reliable low-dimensional visualizations of big datasets, which allows us to perform an intuitive assessment of the quality of the solution. The interested reader may review in the S5 Appendix of S1 File the election of hyperparameters of tSNE. The second part of the clustering, OPTICS, or *Ordering Points to Identify the Clustering Structure* [67] is used as an unsupervised method to discriminate the distinct groups produced by tSNE. OPTICS is a density-based clustering method where clusters are regions in which the objects are dense and are separated by *noise* or regions of low object density, making these approaches to be flexible enough to accommodate clusters of arbitrary shapes and distribution of points. We decided to use tSNE plus OPTICS, instead of only OPTICS, since we empirically checked that the former methodology resulted in better identifiability of clusters of similar networks. Now, the hyperparameters of OPTICS are usually dependent on the dataset, so there is no direct way of setting them. Our approach was to devise heuristics to choose these parameters. The classes produced by OPTICS with this selection of parameters visually match the dense regions of tSNE with coarser separation. The interested reader may review the S5 Appendix of S1 File for details of the heuristics.

#### Description of clusters by classification trees

After obtaining a robust clustering of each dataset, we compute a series of decision trees (DT) to classify the networks into the distinct groups produced by the clustering stage, trained with the same features employed in the clustering, plus the model variables *F*, *ph*, *nh*, *F* * *nh*, *ph* * *F* * *nh* and the mean of the probability of eating for non-hunters. In S6 Appendix of S1 File, the interested reader may review an informal argument for the importance of these features. Now, the classification tree model we use corresponds to that proposed by Loh (2009) [68], since it offers a robust split selection strategy based on selecting the variable with the most significant chi-squared main effect, which redounds in predictive accuracy and model parsimony. Since we look for a robust characterization of the dataset clustering, we compute several trees, each trained with a bootstrap sampling with replacement of size 70% of the dataset, thus it is expected that each training set has approximately half of the set of unique points of the dataset [69]. These computed trees are examined to find the most reliable ones, which we define as those trees that are Pareto optimal for maximization of balanced accuracy [70], which accounts for possible imbalanced classes, and a measure for syntactic stability. We are interested in syntactic stability due to the fact we use the DT representation to draw conclusions about the dataset. Several details regarding the validation of the decision trees may be consulted in the S7 Appendix of S1 File.

## Results

In this section, we report the analysis of model optimal networks, carried out through the pipeline of analysis described in the last section. We review the typical structure of the three types of optima, RV, WEF local optima, and Pareto optimal networks for both criteria, with an emphasis on the behavior of some patterns found in social networks, namely, reciprocity and community structure. In this sense, we have found WEF optima display high clustering and low network segmentation, with no distinguishable modules. Local optima for RV present more diversity, ranging from cooperation exclusive among hunters to networks similar to WEF optima. They may present community structure, but with rather sparse connectivity. Finally, Pareto optimal networks show even more diversity, including either networks similar to the already described local optima, but also new configurations such as distinct cohesive modules around hunters, or high reciprocity between a hunter and non-hunters.

### Welfare optima

#### Summary of distinct types of WEF optima

**Fig 3:**
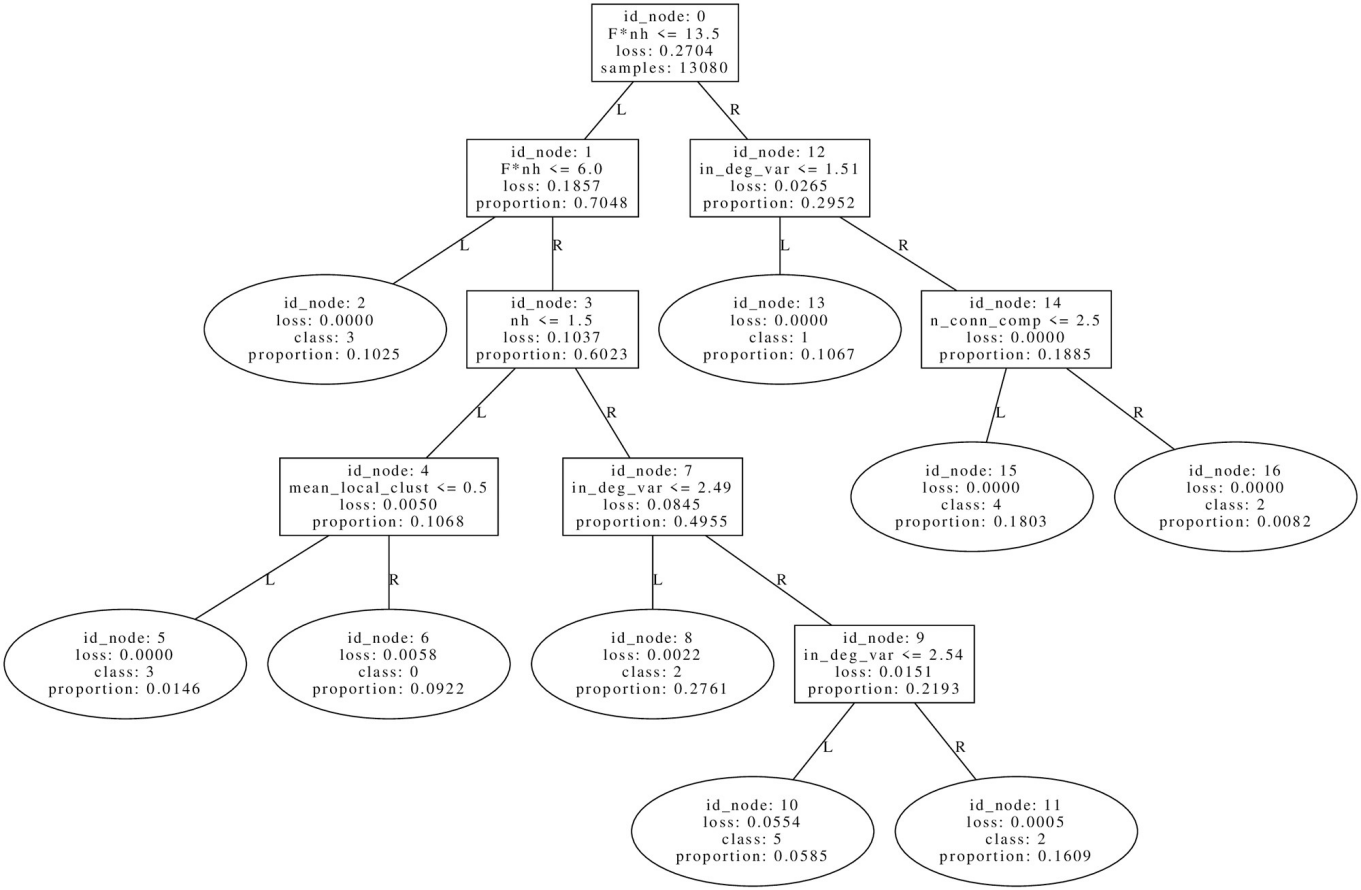
An efficient tree (accuracy = 0.872) to discriminate the clustering labels of Welfare optima. Statistics in the tree are computed on the training set, while average accuracy is computed in the test set. See S3 Appendix of S1 File for the sizes of the datasets used, and Paragraph *Description of clusters by classification trees* for the general procedure of tree construction. See the first paragraph from Section *Welfare optima* for an explanation of the variables displayed in tree nodes.

It is instructive to start by inspecting the following tree of WEF optima in Fig 3, belonging to the accuracy-stability-Pareto optimal set, which has an average accuracy of 0.872 to discriminate the 7 clustering classes. In these trees, the loss corresponds to the Gini coefficient of samples at that node, the predicted class is the class with a maximum number of samples, and proportion is the relative proportion of node samples with respect to the total in the root. This total and the other statistics present in the tree are computed on the training set. The accuracy, on the other hand, is computed on the test set, which is the complement of the training set with respect to the whole WEF dataset of size 18686 (see Table 1 in S3 Appendix of S1 File).

**Fig 4:**
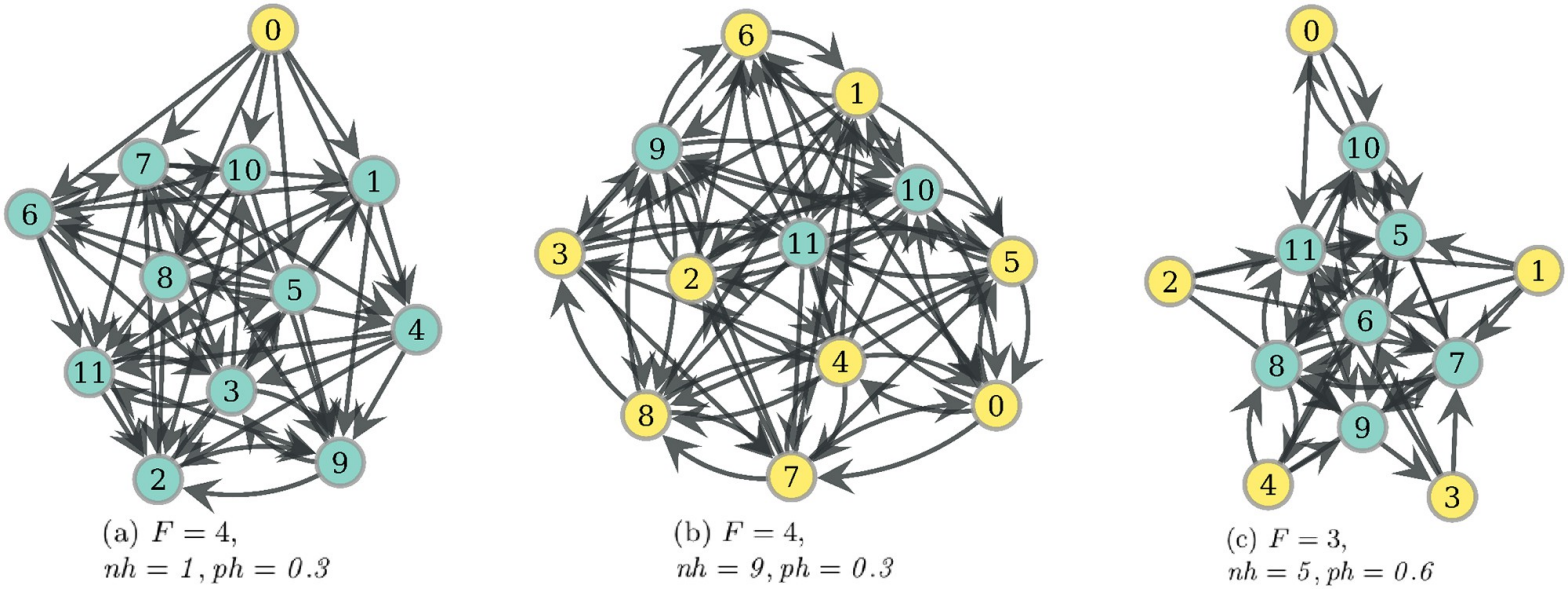
Most central networks of nodes 2 (left), 13 (center) and 16 (right) from tree in Fig 3. Hunters are filled in yellow. Each network specifies the values of model parameters used as input, and for which the respective network is a local minimum.

The first remark is the presence of the condition *F* * *nh* ≤ *13.5* at root, which turns out to be very stable since in each of the 9 distinct trees found at the Pareto efficient tree set, the structure formed by internal nodes of IDs 0, 1, 3 is present. The threshold 13.5 is an artifact from averaging the two most proximal *F* * *nh* values, 12 and 15, and since *F* * *nh* is a product of two integer variables, the condition *F* * *nh* ≤ *13.5* can be equivalently replaced by *F* * *nh* ≤ 12 = *N*. A possible topological marker of model variable *F* * *nh*, which reflects the relative food abundance, is the mean in-degree of hunters, a variable with which there is a significant Spearman correlation of 0.81. Another possible marker is the number of strongly connected components (SCC), variable with which the correlation, in this case, is −0.574. These relationships are due to the fact that optimal WEF networks tend to form a densely connected group of non-hunters that is fed by hunters, except if there is enough food, in whose case there is only 1 strongly connected component where hunters are also fed back. An important exception to this trend in SCC is shown by networks in node 16, where there are only networks with *ph* = *0.6* and a mean of 3.796 SCC. That is, when there is enough food with a high probability of hunting, the network is segmented into distinct SCC’s that provide an egalitarian probability of eating. These structures are shown in the representative networks on Fig 4.

#### General statistics and modularity on WEF optima

**Fig 5:**
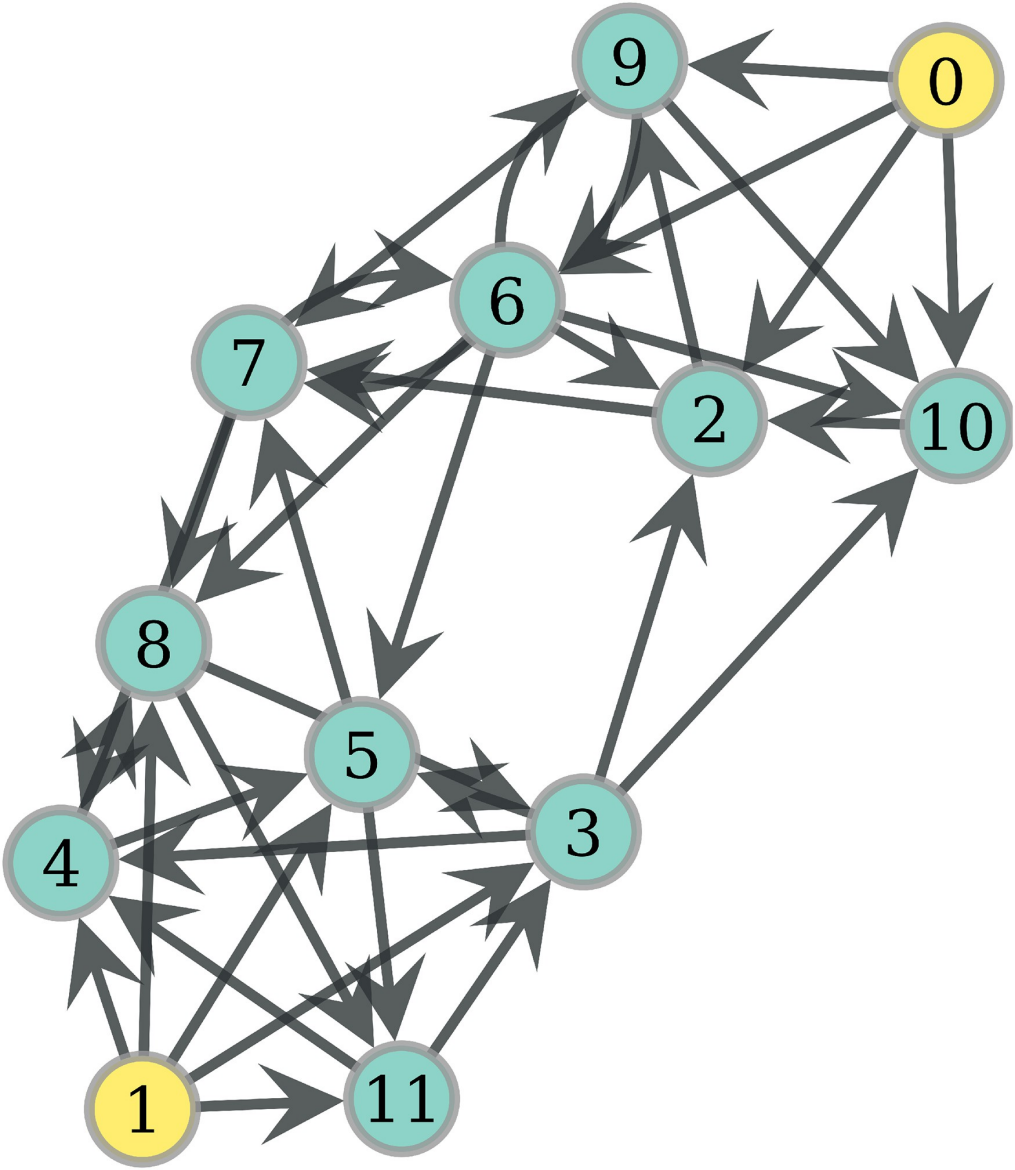
A WEF optima minimizing in-degree variability. Obtained under variables: *F* = 4, *nh* = *2*, *ph* = *0.6*. Hunters filled in yellow.

WEF optima are networks of high clustering, mean out-degree, moderate reciprocity, and low assortativity and modularity, as shown in Table 2, which speaks of one large community with low segmentation. The WEF optimal networks with the largest modularity values, obtain this structure from the special feature of minimizing the standard deviation of in-degree. An example of these networks is in Fig 5 from node 8, having a modularity of 0.305 for the partition {{0, 2, 6, 7, 9, 10}, {1, 3, 4, 5, 8, 11}}.

**Table 2:** WEF optima general statistics.

Feature	Mean	St. dev.
Mean local clustering	0.481	0.167
Mean out-degree	4.763	1.173
Reciprocity	0.232	0.144
Out-assortativity	−0.082	0.044
Modularity	0.042	0.053

### Reduction of variability optima

#### Relation between RV and WEF costs

**Fig 6:**
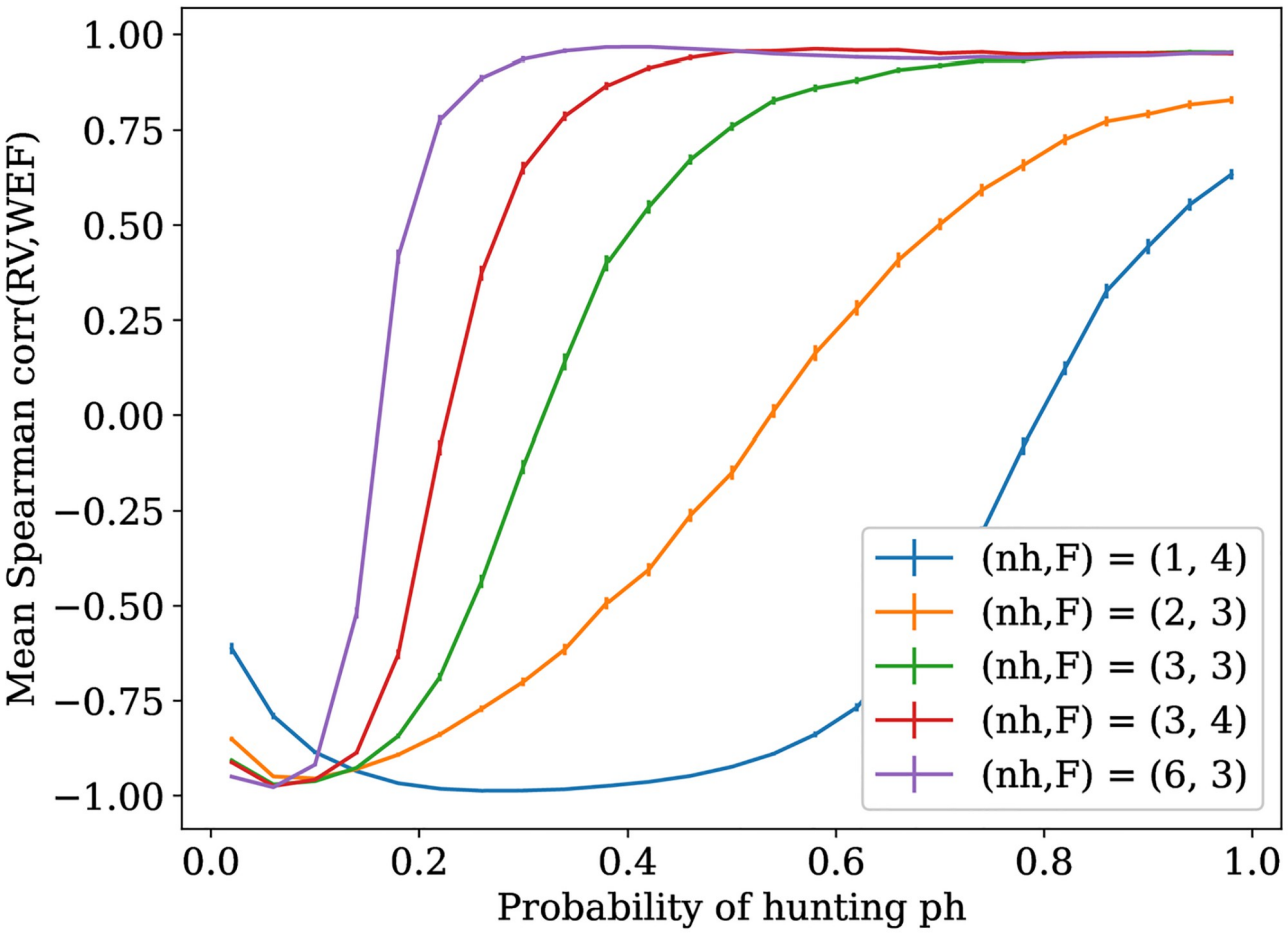
Correlation of costs in function of the probability of hunting. Each curve represents the mean of Spearman correlation between RV and WEF for a specific configuration of *F* and *nh*, and a range of 25 equidistant *ph* values. Each point is the mean of 100 correlation points, each one computed for a uniform random sample of 100 networks. Each mean point is surrounded by the percentile confidence interval using the standard error of the mean with error *α* = 0.05, that is, assuming the sample mean is normally distributed.

We start this section by analyzing the structure of the relation between costs RV and WEF, and derive from it a profile of the distinct networks obtained as RV local optima. We have mentioned in S2 Appendix of S1 File the intuition regarding the expected behavior of RV optima from RV function. That is, for values of *ph* smaller than *pe*⋆, the probability producing the RV maximum, there is an *every man for himself* regime, or free-riding situation where non-hunters just do not receive food and are isolated nodes. Now, for *ph* > *pe*⋆, there is *progressive cooperation*, an increasing trend of feeding non-hunters, particularly when RV reaches its range of flat values. On the other hand, depending on the values of model variables, hunters often share food in order to decrease the *RV* mean. We can visualize this pattern in Fig 6, where we graph the mean correlation values between *RV* and *WEF* for randomly sampled networks and distinct *ph* values.

In this graph, it can be seen that these mean correlation values start approximately at −1 for low *ph* values, which corresponds to the free-riding regime, and in the extent *ph* increases the correlation curves tend to stabilize around values near to perfect correlation. Observe that most curves become increasing functions only for values of *ph* greater than *pe*⋆ = *0.0899*. The fact that greater values of the product *F* · *nh* have a faster convergence to cooperation may be explained, if one assumes a uniform feeding probability, from Equation (7) (S6 Appendix of S1 File), where a greater *F* · *nh* implies greater mean probability of eating, which makes these probabilities closer to the flat RV region.

#### Distribution of arc types and representative RV optimal networks

**Fig 7:**
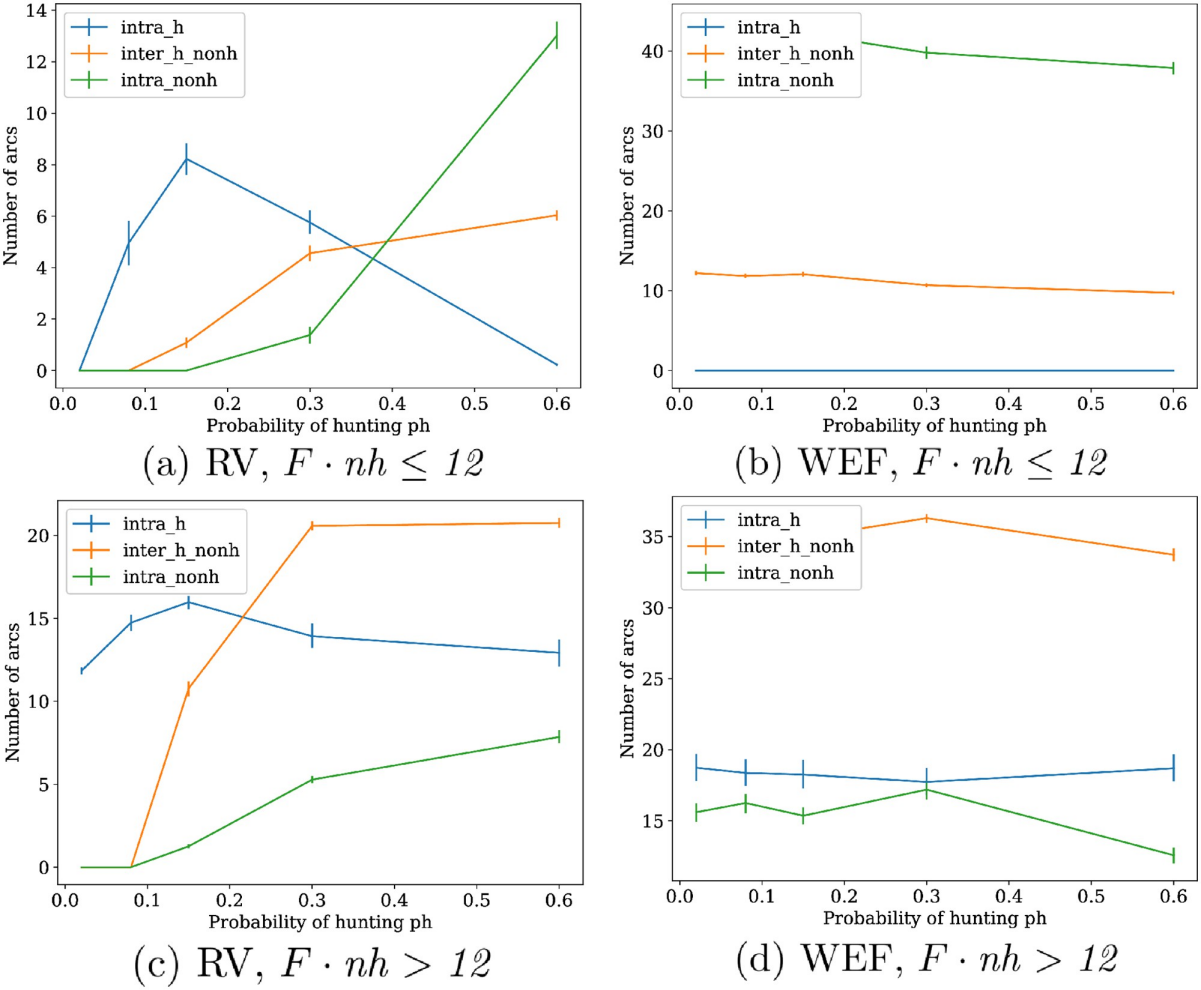
Distributions of arc type for distinct optima and food supply condition. The three types of arc graphed (which do not consider arc direction) are: connecting two hunters (*intra_h*), connecting two non-hunters (*intra_nonh*), and connecting a hunter with a non-hunter (*inter_h_nonh*). Each number of arc type average is computed for every sampled *ph* value, and surrounded by a percentile bootstrap confidence interval of error *α* = 0.05 and 1000 bootstrap samples of 70% of dataset. See the paragraph *Summary of distinct types of WEF optima* from Section *Welfare optima*, for the result justifying the use of condition *F* · *nh* ≤ *N* to aggregate datasets.

This pattern of costs behavior is reflected also in the distribution of distinct types of arcs. If we separate arcs in three groups depending on whether they connect, without arc direction: two hunters, a hunter and a non-hunter, and two non-hunters, we obtain the following distribution of the number of arcs depicted in Fig 7. It can be seen in the figure that, in the two smallest *ph* values, non-hunters are isolated in RV optima, which is part of the so-called every-man-for-himself phase. If *ph* increases, the relative numbers of arcs gradually converge to the same ordering displayed by WEF optima, which occurs for lower values of *ph* if *F* · *nh* > *12*. This pattern is consistent to what is displayed in Fig 6.

**Fig 8:**
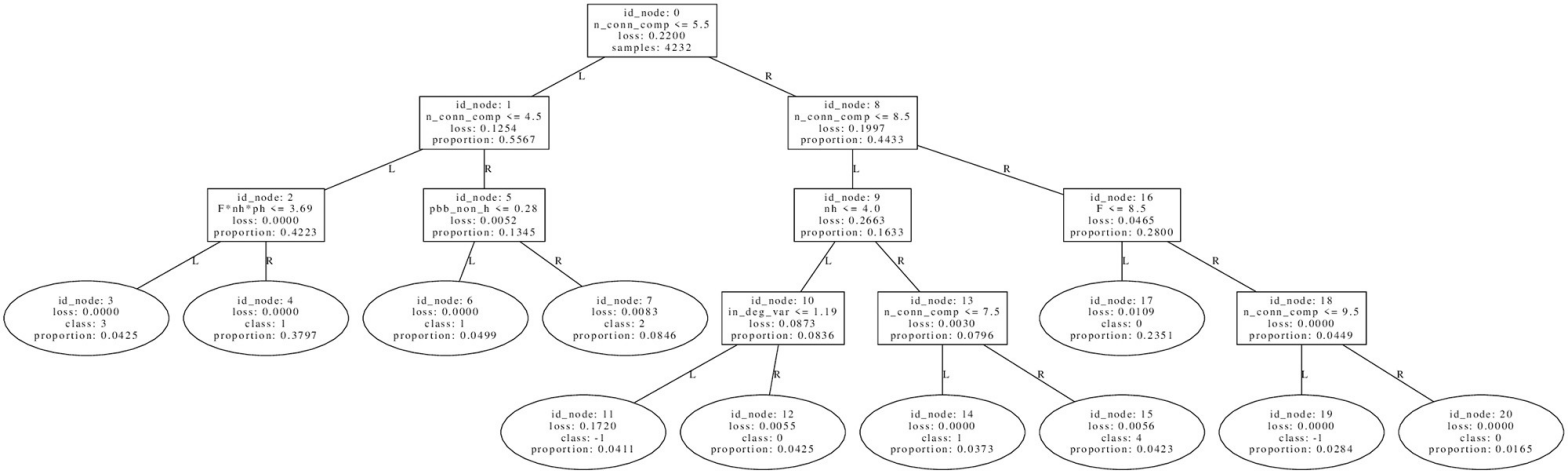
An efficient tree (accuracy = 0.907) to discriminate the clustering labels of RV optima. Statistics in the tree are computed on the training set, while average accuracy is computed in the test set. See S3 Appendix of S1 File for the sizes of the datasets used, and Paragraph *Description of clusters by classification trees* for the general procedure of tree construction. See the first paragraph from Section *Welfare optima* for an explanation of the variables displayed in tree nodes.

**Fig 9:**
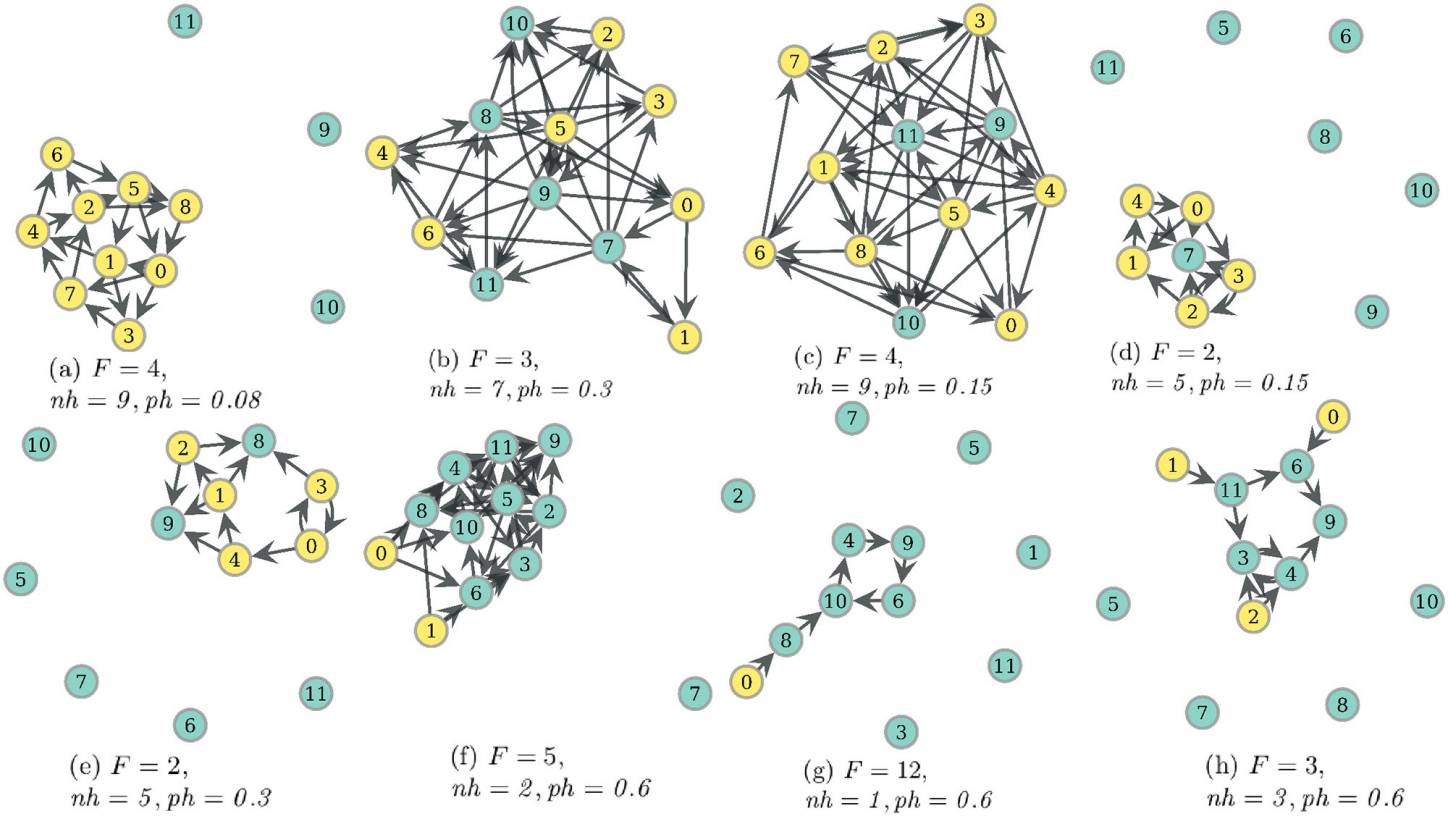
Example networks from distinct DT leaves of the tree in Fig 8. From left to right, and from top to bottom: (a) Leaf 3 (most central), (b) Leaf 4, (c) Leaf 4, (d) Leaf 15, (e) Leaf 17, (f) Leaf 7 (most central), (g) Leaf 19 (most central) and (h) Leaf 17. Each subcaption displays values of the condition under which these optima were obtained. Hunters filled in yellow.

The distinctive profiles of the number of arcs from these graphs may be used to identify representative networks obtained as RV optima. In Fig 8, a decision tree that discriminates the classes of RV optima with an accuracy of 0.907 is displayed, together with representative networks from some of its leaves in Fig 9. In the left part of the tree, there are the networks with a lesser number of strongly connected components, which are networks with greater values of *F* · *nh*. The networks from Fig 9(a)–9(c), show the prototypical behaviors shown in Fig 7(c), which are, respectively, the absence of connectivity for non-hunters if *ph* is low, a network with WEF-like topology of minimization of in-degree variability if *ph* ≥ *0.3*, and a transition between the two regimes similar to the last one, but where hunters have more intra-connectivity rather than inter-connectivity.

Now, some of the patterns from Fig 7 (a) are displayed in Fig 9(d)–9(f). In the first of them, there is a network from Leaf 15, where networks have arcs mainly among hunters, and may present some few connections to non-hunters. The networks of this kind are among those in RV optima having the greatest proportions of reciprocated arc pairs, usually only between hunters. This is used as a strategy to lower the high RV values in this range of *ph* values (*ph* = *0.15*). Then, in Fig 9(e), there is more connectivity to non-hunters, but it is still comparable to that between hunters. Finally, Fig 9(f) displays a network with the cooperative regime on *ph* = *0.6* where intra-non-hunters connectivity surpasses the number of connections to hunters, and these display a minimal intra-connectivity. The case when *ph* = *0.6* and there is just one hunter, as in Fig 9(g), is special. These networks present a one-way walk with no forks, since a fork lowers the probability of eating with respect to the no-fork situation, increasing the RV cost. If there are isolated nodes, as is observed in most optima obtained from an evolutionary optimization, there is a big difference in the probability of eating between connected and isolated nodes, which increases WEF cost. This affects the Spearman correlation between *ph* and WEF in the whole dataset, which is 0.313. If we restrict to those data where *nh* > *3*, this correlation lowers to −0.319, and to −0.73 if *nh* > *6*, consistent with Fig 6.

#### Modularity on RV optima

We close this section by remarking on the behavior of modularity on this type of optima. In general, RV optima are networks of low modularity, see Table 3 for its general statistics, but in fact, high modularity may be observed. This high modularity is a by-product of survival or network arrangements for enhancement of probabilities of eating, as can be pointed from its correlation with *F* · *nh* which is −0.359. However, the relationship is stronger for higher *ph* values, since restricting to *ph* > *0.2* gives a correlation of −0.474. This higher modularity is usually more related to a segmentation in the network than to the presence of cohesive groups of nodes. An example of this is the same network from Fig 9(g), which has a modularity of 0.208 for the partition {{0, 1, 2, 3, 5, 7, 8, 11}, {4, 6, 9, 10}}. Another example is that of Fig 9(h) having a modularity of 0.32 for partition {{0, 6, 8, 9}, {1, 10, 11}, {2, 3, 4, 5, 7}}.

**Table 3:** Modularity general statistics on RV optima.

Percentile 0.5	Percentile 25	Median	Percentile 75	Percentile 99.5
0	0.029	0.08	0.145	0.48

### Multi-objective optima

#### Overview and scope of this analysis

In this section we show some of the key social networks patterns that can be recovered in Pareto optimal networks from the simultaneous optimization of RV and WEF criteria, which we will refer to as PF (due to their criteria values being in the Pareto Front) optima. We start by showing graphs of average behaviors displayed by the distinct type of optima, highlighting the most relevant differences among them. We then illustrate these behaviors by depicting some representative Pareto optimal networks and conclude with a brief analysis. Since there is a wide spectrum of Pareto optimal networks, we restrict the study to the most interesting case of *F* · *nh* ≤ *N*. See the paragraph *Summary of distinct types of WEF optima* from Section *Welfare optima*, for the result justifying the use of this last condition. Most types of optima networks from the context *F* · *nh* > *N* appear also as optima in the former case. The interested reader may review the trees of the case *F* · *nh* >*N* and associated optimal networks, in the documentation for running the code from the repository provided for this purpose [71].

#### Community structure on PF optimal networks

**Fig 10:**
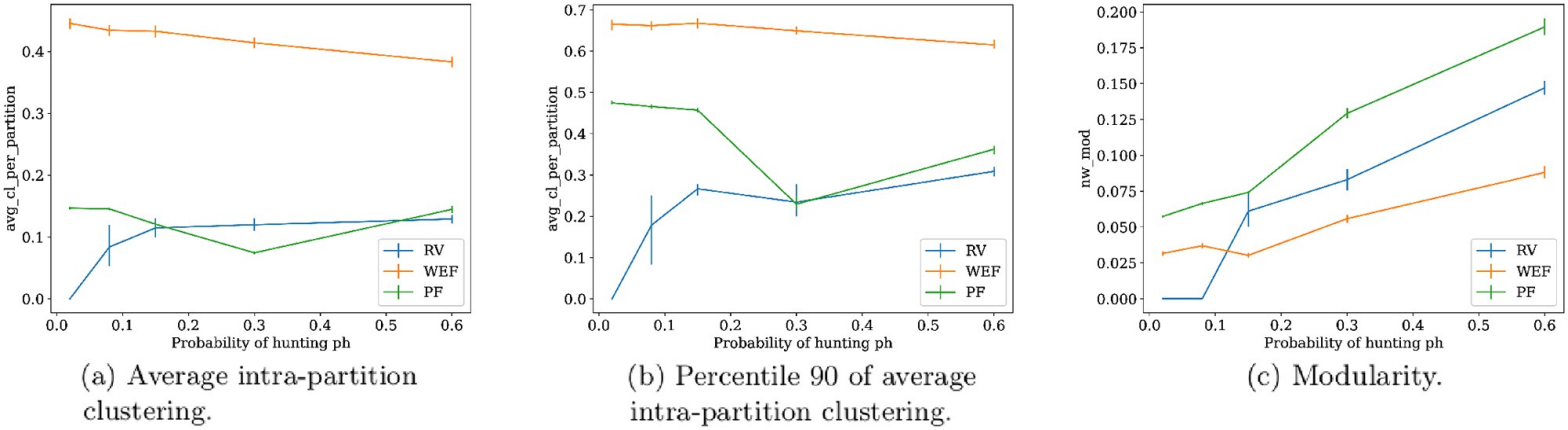
Distributions of different measures of community structure for each type of network optima. From left to right: Average intra-partition clustering (left), percentile 90 of average intra-partition clustering. (center), and mean modularity (right), for each type of network optima. Average intra-partition clustering (AIC) is the mean of local clustering computed in each subnetwork induced by the modules of the partition maximizing modularity. These statistics are computed on each of the 500 bootstrap samples with replacement of size 70% from dataset, used to compute percentile confidence intervals with error *α* = 0.05 for each statistic.

One of the main results is displayed in Fig 10, where there are two measures of community structure, the already mentioned network modularity, and a new measure called *average intra-partition clustering*. This measure corresponds to the average of local clustering over the subnetworks induced by each of the modules of the partition maximizing modularity found by the Monte Carlo algorithm. Thus, intra-partition clustering is a measure of the cohesiveness of the partition maximizing modularity. A first remark to note is that, though maximum values of this measure are obtained by WEF optima, this pattern is more a product of the high connectivity across the whole network found in this type of optima, than to the presence of clear cohesive modules. This observation is supported by the low values of modularity exhibited by WEF optima, see Fig 10(c), and is coherent with the hypothesis that the partitioning found in this optima obeys more to the random nature of the Monte Carlo algorithm.

**Fig 11:**
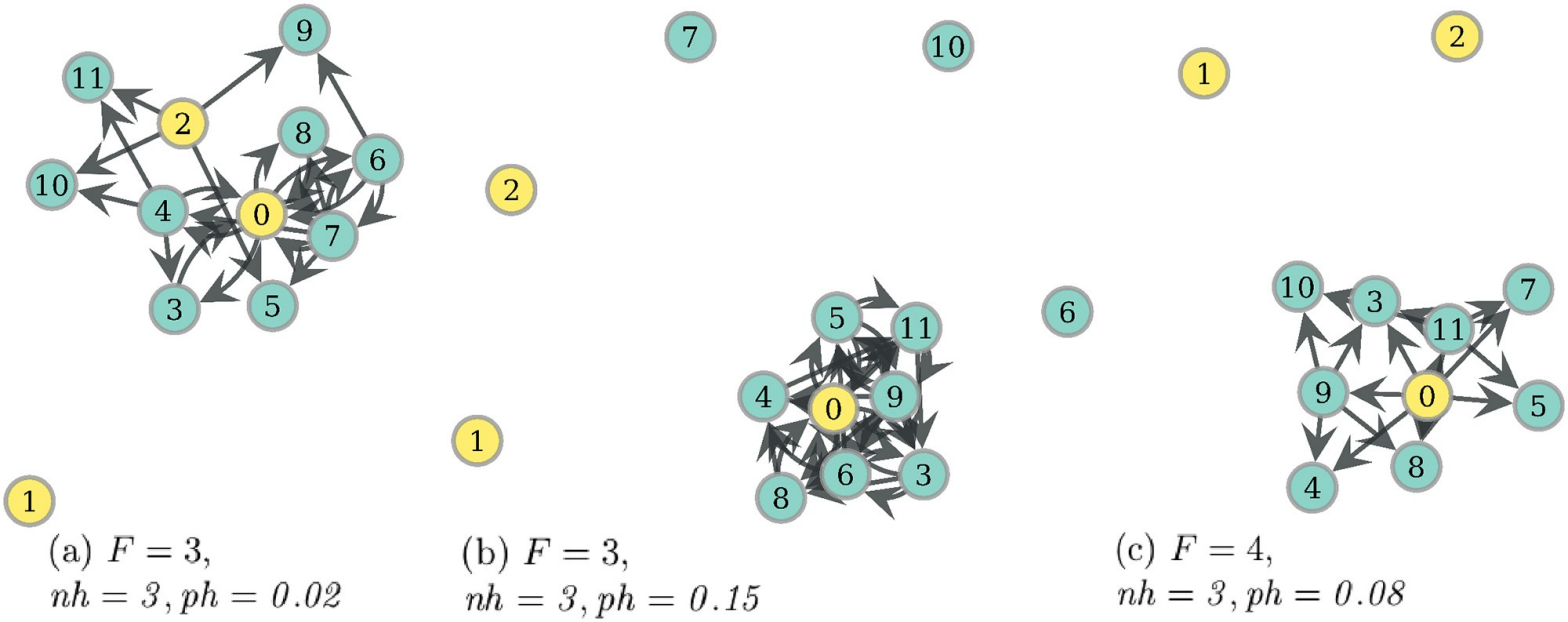
Representative PF networks of regime *ph* ≤ *0.15*. Model variables under which these Pareto optimal networks were obtained are displayed under each network. Hunters are filled in yellow. To obtain representative networks, these were chosen as the most central from leaves of efficient trees with a relative proportion higher than 8%. To see the trees, consult the S8 Appendix of S1 File.

**Fig 12:**
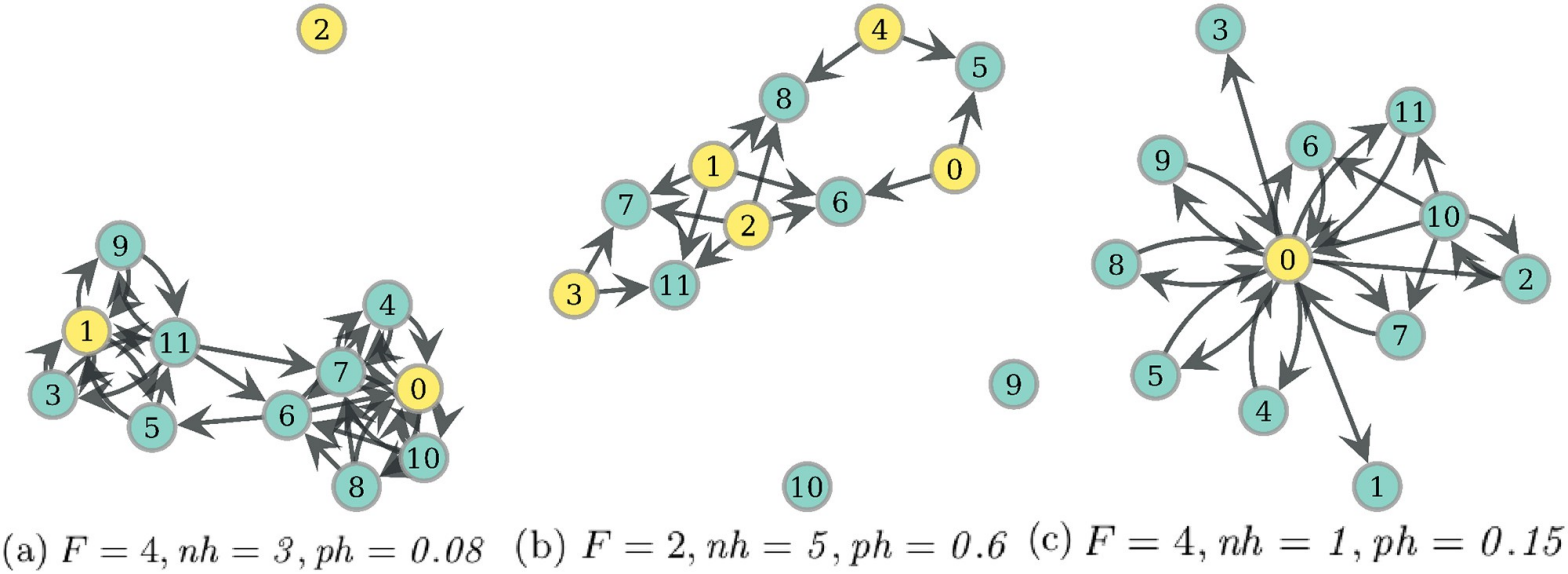
Representative PF networks with some social network pattern. Model variables under which these Pareto optimal networks were obtained are displayed under each network. Hunters are filled in yellow. Average intra-partition clustering (AIC) is the mean of local clustering computed in each subnetwork induced by the modules of the partition maximizing modularity. From left to right: network (a) has a modularity of 0.392 and AIC of 0.454, network (b) has a modularity of 0.221 and AIC of 0, while network (c) has a modularity of 0 and AIC of 0.029. Additionally, this last network has 7 reciprocated arc pairs between hunters and non-hunters.

Now, if one inspects the mean values of average intra-partition clustering (AIC) of PF optima, one notes that they are not too distinct from those of RV (Fig 10(a)). As an example, for the observed PF networks with *ph* ≤ *0.15*, the mean is 0.148 and the median is 0.056, and one can find typical networks such as those in Fig 11(a)–11(c), with AIC values of 0.083, 0.313 and 0.023, respectively. However, if one looks at the highest values of this measure in Fig 10(b), it is noted PF gets higher values than RV, for *ph* ≤ *0.15*. This pattern is due to networks such as that of Fig 12(a), where there are two modules of connected nodes around distinct hunters, which is reflected in a value of 0.454 for intra-partition clustering. On the other hand, if community structure is measured with modularity index, it can be seen in Fig 10(c) that PF mean increases with *ph*, and it is higher than that of single optima networks. This behavior is influenced mainly by networks that indeed can exhibit relatively large modularity, but with weakly connected modules, similar to the architecture of some RV optima. Fig 12(b) displays an example of this type of PF optima.

#### Reciprocity on PF optimal networks

**Fig 13:**
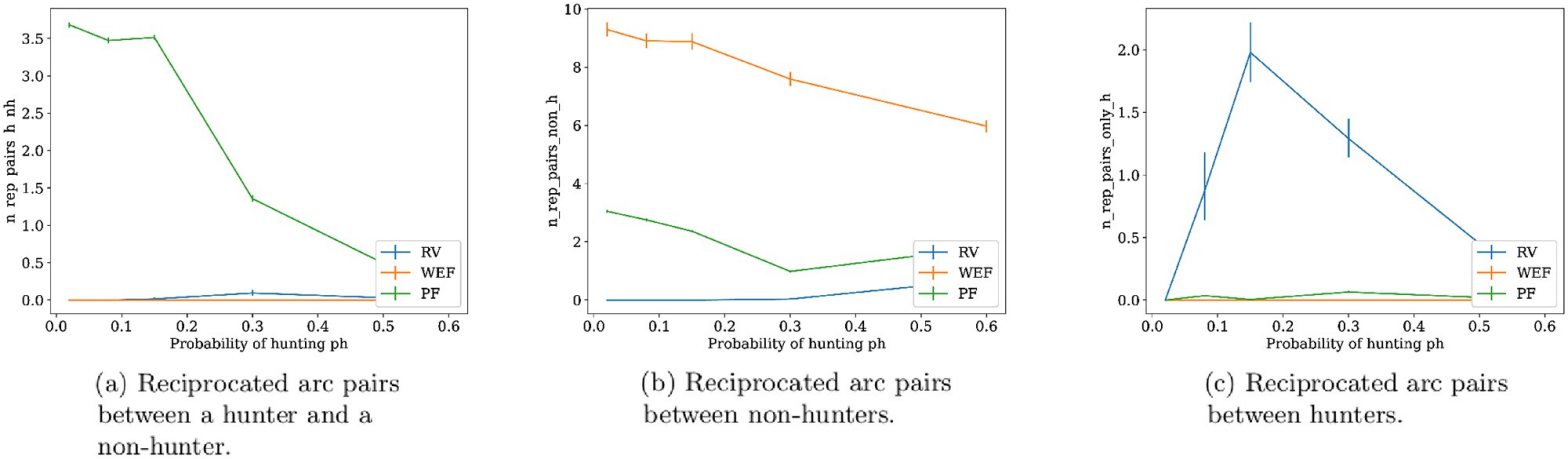
Distributions of different types of reciprocated arc pairs for each type of network optima. From left to right: Mean number of reciprocated pairs between a hunter and a non-hunter (left), mean number of reciprocated pairs between non-hunters (center), and mean number of reciprocated pairs between only hunters (right). These means are computed on each of the 500 bootstrap samples with replacement of size 70% from dataset, which are used to compute percentile bootstrap confidence intervals with error *α* = 0.05 for each statistic.

**Fig 14:**
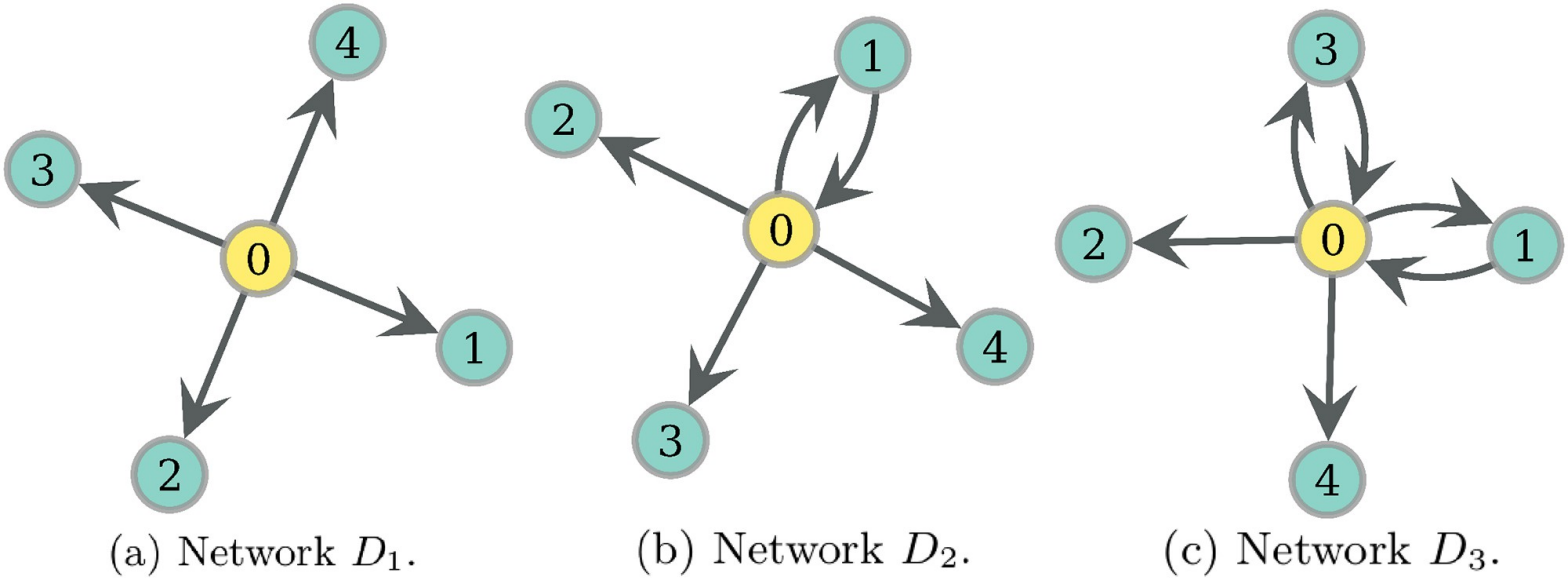
Three networks with increasing number of reciprocated arcs between hunter and non-hunters. We assume there is 1 hunter, node 0, *ph* = *0.08* and *F* = 4. Network *D*_1_ gets costs *RV* = 0.261 and *WEF* = 0.024, *D*_2_ obtains *RV* = 0.315, *WEF* = 0.02 and *D*_3_, *RV* = 0.332, *WEF* = 0.018.

The behavior of the other social network pattern we study, reciprocity, can be appreciated in Fig 13. There it can be seen that, particularly, the presence of reciprocated arcs between a hunter and non-hunters is very salient on PF networks compared to the other optima. This pattern is observed in networks such as that of Fig 12(c). On the other hand, the presence of reciprocity among non-hunters in Fig 13(b), is associated with networks similar to that of Fig 11(b), which for example has 5 reciprocated arcs among non-hunters. The intuition for the presence of reciprocated arcs between a hunter and non-hunters, as well as other patterns found on PF optima, is given by the notion of dominance, which defines the Pareto optimal set, and will be explained next through the example of Fig 14.

#### Rationale of traits present on PF networks

Since *ph* = *0.08* for every network in Fig 14, in network *D*_1_ each non-hunter eats with equiprobability 0.02. If we add one reciprocated arc pair on node 1, we obtain the network *D*_2_ in Fig 14. And since *F* = 4, now each non-hunter in *D*_2_ eats directly from the hunter, and also from the food that first went through node 1. Thus, node 1 eats with probability 0.02 while every other non-hunter eats now with probability 0.035. This reduces the WEF cost, and since we are in this range of low probabilities, the mean of RV costs increases. See the exact values in the Figure description. If now we add a new reciprocated arc pair to node 3 we get the network *D*_3_ in Fig 14, where now node 1 eats additionally from the indirect route through 3. This implies that every non-hunter in *D*_3_ eats with a probability of 0.035, which again increases RV and lowers WEF. Thus, for this condition of model variables and these simple networks, the presence of each of these reciprocated arc pairs delivers a non-dominated network, since the deletion of this trait cannot simultaneously lower both costs, as we have seen.

#### Probability of eating

**Fig 15:**
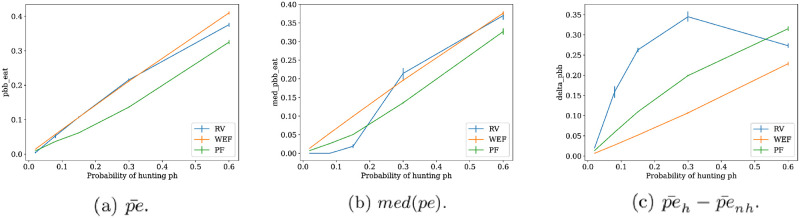
Display of three variables associated with the probability of eating for each type of network optima. From left to right: mean probability of eating, median probability of eating, and difference in means of the probability of eating between hunters and non-hunters. In each graph, the mean of each statistic is drawn surrounded by percentile bootstrap confidence intervals with error *α* = 0.05, computed on each of the 500 bootstrap samples with replacement of size 70% from the dataset.

As a final observation, we look at the behavior of the probability of eating in a network. The mean probability (over network nodes) is on Fig 15(a), and it turns out that PF obtains lower means over dataset samples. This is due to the high prevalence of networks with one hunter and reciprocated arcs between hunter and non-hunters, which usually get low mean probabilities, due to low food availability. If now we look at the median probability on Fig 15(b), it is observed that PF offers a greater probability than RV on low *ph* values. This is because in PF there are networks similar to WEF minima that deliver better probabilities of eating that do not leave isolated nodes as with the RV optima. This is confirmed by Fig 15(c), which shows the greatest differences between hunters and non-hunters are in the RV optima, and by the remarkable fact in Fig 15(b) that WEF optima shows the greatest efficiency in the probability of eating in the whole range of *ph*.

## Discussion

In this section, we discuss the main contributions of our model in relation to previous works, possible implications of our results, and avenues for future research.

### Condition for community network structure

Communities given by cohesive network modules appear in food sharing networks optimized to provide egalitarian access to food resources jointly with reduced individual risk. This result is obtained under conditions of uncertainty and scarcity in the food supply (see Figs 10(b) and 12), which suggests their adaptive value for this context. The simultaneous optimization of the two goals is necessary since RV optima may present community structure but with rather sparse connectivity (see Fig 9), while WEF may present communities under the special case of degree dispersion minimization (see Fig 5). These PF networks are resemblant to the food-sharing networks observed by Dyble et al (2016) [21], where there are cohesive groups corresponding to households provisioned by an adult couple, and a set of households forms a cluster with a small number of inter-household connections. In our model, the cohesive groups correspond to consumers, or non-hunters, grouped around one or more hunters. This result suggests that not only the camp level, which groups several clusters, is functionally associated with risk reduction reciprocity as argued by Dyble et al (2016), but also the hierarchically lower levels of households and clusters may have been related evolutionarily to some resource distribution optimality that includes the egalitarian access to resources. This idea is consistent with some studies suggesting that many kinship configurations may be a product of sharing patterns [12, 43, 44].

The claim that the two minimization criteria are required for the emergence of cohesive communities is reinforced by the condition of uncertain food generation, not only because it is under this regime where communities are obtained (Fig 12), but also because when food supply is safer, the two criteria become indistinct to the dense optimal WEF networks with low modularity (Fig 9(f)). The fact that sharing is directed mainly towards large and uncertain packages of food is acknowledged by several empirical studies [4, 40, 41, 46]. The necessity of the confluence of both the individual and group interests has also been pointed out in the simulation agent-based study by Briz i Godino et al (2014) [72] which models the cooperative public calls through smoke signals of a hunter-fisher-gatherer society conditional on the exceptional accumulation of fish. The model shows that a low weight on social capital relative to individual consumption of meat in the agents’ fitness, is enough to obtain most society cooperating as the most likely outcome, but this result may be reversed when this weight is absent. This social capital component of reproductive fitness seems to be a plausible mechanism according to Chaudhary et al (2016) [73], where it is reported that relational wealth, or the cooperative relationships an individual has, provides advantages in buffering food risk, body mass index, fertility, and is partially heritable.

Finally, the result that a stringent resource availability would be necessary for the evolution of cooperation is supported by another agent-based model named *Cooperation under resource pressure* (CURP) by Pereda et al (2017) [74]. This work shows that under moderate survival stress, populations of agents self-organize in an indirect reciprocity system consisting in sharing the part of the resource that is not strictly necessary for survival, achieving to collectively lowering the chances of starving. Additionally, a high-stress regime turns into unstable behavior, where the population constantly searches for survival strategies, while low-stress does not exert selection and strategies remain almost constant and randomly drifting. Since the variables in our model may be understood as a form of resource-pressure, and even a parallel may be established between the two models’ variables (*prob-resource* to *ph*, and *min-energy* to *F*), the results of our model roughly resemble those of CURP, taking into account that CURP is not a social networks model, and that ours is not an agent-based evolutionary model. This aclaration allows us to understand the fact that our model does not produce a clear distinction between moderate and high-stress scenarios in the sense CURP does. Our model does not incorporate a notion of dynamics like evolutionary models, but rather gives a fixed optimal organization under certain conditions. On the other hand, CURP does not make a distinction between hunters and non-hunters, and all agents can produce food.

### Network structures promoting egalitarian resource distribution

Welfare optimal networks (WEF) display high connectivity but low network segmentation, where each hunter is connected to one big, homogeneous and dense group of non-hunters, see Fig 4. On the other hand, networks minimizing individual risk (RV) range from cooperation exclusive among hunters, to networks more similar to welfare networks when the food supply is safer, see Fig 9. These networks are very similar to those tested in the lab experiment by Chiang (2015) [48], where it is found that network structures linking agents with discrepant income levels promote more egalitarian distributions by motivating the rich agents to share their incomes with the poor. Particularly, the network *SF_negative* from the experiment is very similar to the undirected version of WEF optima from our model. On the contrary, networks where similar income agents are linked evolve to more unequal income distributions, which is also consistent with the RV optima in stringent resource conditions where hunters are only connected among themselves. This result is consistent with the view that social networks structured originally according to ecological considerations, may have created the environment in which prosocial tendencies and equity response elicitation as those explored in the experiment, evolved along human history [49, 50].

Finally, the fact that many WEF optima minimize degree variance is consistent with results of a previous simulation of need-based transfers evolution model showing that degree variance is anti-correlated with survival rate [75]. The minimization of in-degree variance is another pathway to cohesive communities found in our model, see Fig 5. This scenario may be the idealized case resultant of some other more realistic constraint as, for example, an upper bound on the number of connections an agent may have. This last idea has been proposed by the *social brain hypothesis* [76], which states that there are cognitive constraints on the number of face-to-face social interactions a human may have, where these limits would be inversely related to the emotional closeness of the bond. Thus, for example, the number of close friends one could maintain is limited to five. The evolutionary perspective suggests, again, that these cognitive constraints on social relationships may have been related originally to some kind of resource distribution optimality.

### Distinct patterns of reciprocity

We have obtained distinct distributions of reciprocity among hunters and non-hunters, in the three types of optimal networks we study, which gives a broader picture to the usual notion that reciprocity is driven by the minimization of food production uncertainty [28, 77]. We only got high reciprocity under the reduction of variability criterion (RV) for reciprocal exchanges among hunters, see Fig 13(c). On the other hand, Pareto optimal networks (PF) maximize mean reciprocity between hunters and non-hunters, and Welfare (WEF) maximizes mean reciprocity among non-hunters, see Fig 13. These distinct distributions may suggest that the relative importance of every criterion guiding food transfers depends critically upon the context, which in turn may be a result of population size and division of labor.

One possibility is that for large communities, where hunters are only a small fraction of the whole population, there are hunters that reciprocally exchange food to minimize risk, and after that, new exchanges take place within subcommunities fed by each of these hunters, that may be driven to a greater or lesser extent by welfare considerations. This is not far from what has been reported for some hunter-gatherer societies, where there are two systems or stages of sharing: in the first, sharing occurs among participants in the cooperative effort of food acquisition as a form of labor reward, and then, in the secondary distribution each individual that received shares redistributes his or her share to families that did not participate [51]. This is consistent with the claim that exchanges motivated by need or welfare, would be more common within a household [20, 78], and with more recent observations reporting that Hadza men consumed a substantial amount of food while out of camp foraging [79], and that returning to camp empty-handed is indicative that he failed to produce enough surplus to share. This may suggest a greater relative weight of individual starvation risk minimization over welfare motivation in the first stage of sharing.

### Implications for evolutionary and economic models of social dynamics, and future work

Though our model is not evolutionary in its formulation, its results suggest that motives not usually modeled by these approaches, such as the egalitarian group access to critical resources for survival, may be an important driver for social network formation. Typical evolutionary models of food-sharing [38] rely on the assumption of maximization of a function of subjective preferences, usual in economic models of network formation [80], which highlights an exclusive role of individual choice and may impose high cognitive requirements. We claim that evolutionary approaches may benefit from a wider repertoire of assumptions including a resource distribution perspective, the modeling of survival needs, and the explicit inclusion of the group level of analysis.

Our model looks for capturing the transfers of finite resources subject to uncertain and scarce production, and their effect on optimal network organization. Therefore, its insights may potentially be applied to other situations imposing an analog structure of restraints. For example, since parents need to spend most of their time raising their offspring, maybe the behavior of cooperative breeding is dependent on the uncertain generation of scarce free time available for the care of non-descendant children. On the other hand, some works have argued [32, 41] about the usefulness of using a diminishing marginal returns function over the exchanged quantities of food, which may be a symptom of the underlying phenomenon of satisfaction of needs [42]. Our model is based on an original formulation of starvation risk relying upon the expected number of success runs of a certain length in a sequence of trials to implement the hunger need over time. Our model may contribute a formal framework to proceed in this discussion. Finally, the explicit inclusion of group level of analysis may be implemented through the multi-level selection model [81, 82] that incorporates as evolutionary units the individuals, as well as the groups they form. A mapping from individual fitness to starvation risk, and from group fitness to egalitarian group access to resources may be studied in future work. A model like this may serve to shed light on the complex interactions between developmental, biological, social, economic and cultural factors influencing social networks formation. More direct extensions of our model would be, for example, more complex protocols of food-sharing allowing for preferential exchanges according to agent closeness, correlations in production of food [77], or others. The modular character of the model would allow making changes at several levels.

## Conclusion

We have presented and analyzed a formal network optimization model inspired by a food-sharing dynamic, that can recover some typical patterns found in social networks. Specifically, we have formalized two main drivers for food-sharing: the reduction of individual starvation risk and the care for the general welfare of agents, and have shown using evolutionary algorithms and data analysis techniques, three main findings, plus a methodological contribution.

- Communities of cohesive network modules appear in food sharing networks optimized to provide egalitarian access jointly with reduced individual risk. This result is obtained under conditions of uncertainty and scarcity in the food supply, which suggests their adaptive value for this context. The simultaneous optimization of the two goals is necessary for this outcome. These networks are resemblant to the food-sharing networks observed by Dyble et al (2016) [21], where there are cohesive groups corresponding to households provisioned by an adult couple, and a set of households forms a cluster with a small number of inter-household connections. In our model, the cohesive groups correspond to consumers, or non-hunters, grouped around one or more hunters. This result suggests that the organization of nuclear families described by Dyble et al (2016) [21] may have been evolutionarily functional for resource distribution optimality.
- Welfare optimal networks (WEF) display high connectivity, but low network segmentation, where each hunter is connected to one big, homogeneous and dense group of non-hunters. On the other hand, networks minimizing the individual risk (RV), which range from cooperation exclusive among hunters to networks more similar to welfare networks when food supply is safer, may present community structure but with rather sparse connectivity. These networks are very similar to those tested in the lab experiment by Chiang (2015) [48], where it is found that network structures linking agents with discrepant income levels promote more egalitarian distributions by motivating the rich agents to share their incomes with the poor. Particularly, network *SF_negative* from the experiment is very similar to the undirected version of WEF optima from our model. On the contrary, networks where similar income agents are linked evolve to more unequal income distributions, which is also consistent with RV optima in stringent resource conditions where hunters are only connected among themselves. This result is consistent with the view that social networks structured originally according to ecological considerations, may have created the environment in which prosocial tendencies and equity response elicitation as those explored in the experiment, evolved along human history [49, 50].
- We have obtained distinct distributions of reciprocity among hunters and non-hunters, in the three types of optimal networks we study, which gives a broader picture of the usual notion that reciprocity is driven by the minimization of food production uncertainty [28], and that may be consistent with some empirical reports [51] on how sharing is distributed in waves, first among hunters, and then hunters with their families.
- As a final contribution regarding methodology, our model is based on an original formulation of survival risk relying upon an estimate of the number of success runs of a certain length, in a sequence of Bernoulli trials. We believe our model may contribute a formal framework to proceed in this discussion regarding the principles guiding food-sharing network formation. Additionally, we employ an original pipeline of state-of-art clustering algorithms to analyze the multiple network optima of our model that may be of interest.

Our model suggests that evolutionary accounts of food sharing may benefit from including a resource distribution perspective, the modeling of survival needs, and the explicit inclusion of the group level of analysis, for example, as a level where selection also operates, autonomously from the individual level. Future work based on this model may contribute to a better understanding of the complex interaction of factors affecting the formation of social networks, and human natural history.

## Supporting information

S1 Fig Two non-isomorphic networks with the same cost for *F* = 3. Networks *D*_1_ (left) and *D*_2_ (right). Hunters {0, 3} are filled in yellow.
(TIF)

S2 Fig An efficient tree (accuracy = 0.747) to discriminate the clustering labels of PF optima on *ph* = *0.02*. Statistics in the tree are computed on the training set, while average accuracy is computed in the test set. See S3 Appendix of S1 File for the sizes of the datasets used, and Paragraph *Description of clusters by classification trees* for the general procedure of tree construction. See the first paragraph from Section *Welfare optima* for an explanation of the variables displayed in tree nodes.
(TIF)

S3 Fig An efficient tree (accuracy = 0.734) to discriminate the clustering labels of PF optima on *ph* = *0.15*. Statistics in the tree are computed on the training set, while average accuracy is computed in the test set. See S3 Appendix of S1 File for the sizes of the datasets used, and Paragraph *Description of clusters by classification trees* for the general procedure of tree construction. See the first paragraph from Section *Welfare optima* for an explanation of the variables displayed in tree nodes.
(TIF)

S4 Fig An efficient tree (accuracy = 0.845) to discriminate the clustering labels of PF optima on *ph* = *0.08*. Statistics in the tree are computed on the training set, while average accuracy is computed in the test set. See S3 Appendix of S1 File for the sizes of the datasets used, and Paragraph *Description of clusters by classification trees* for the general procedure of tree construction. See the first paragraph from Section *Welfare optima* for an explanation of the variables displayed in tree nodes.
(TIF)

S1 File (ZIP)
