# Supplementary figures and images for: Modularity of food-sharing networks minimises the risk for individual and group starvation in hunter-gatherer societies
Source: PLoS One. 2023 May 10;18(5):e0272733. doi: 10.1371/journal.pone.0272733 (PMC10171659; doi:10.1371/journal.pone.0272733)

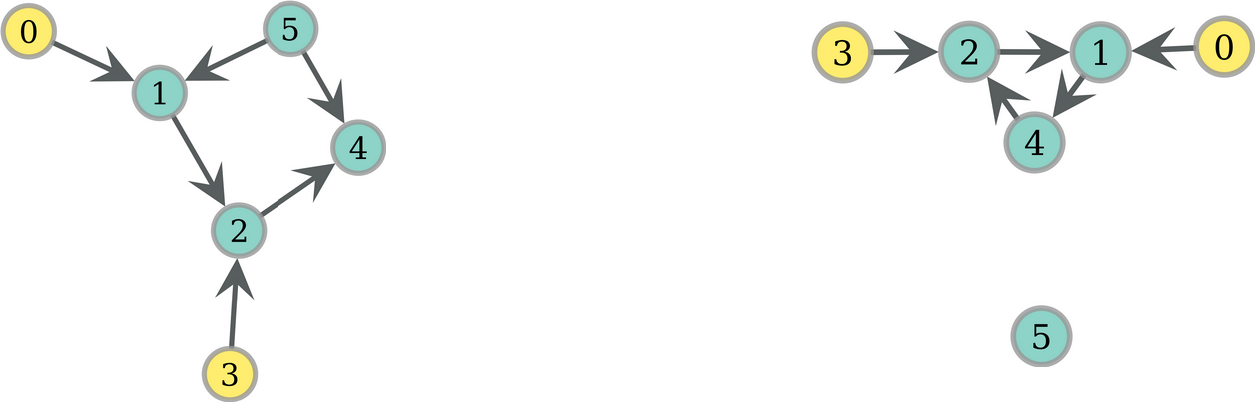

Supplement: S1 Fig — Networks D1 (left) and D2 (right). Hunters {0, 3} are filled in yellow. (TIF) [file pone.0272733.s001.tif]

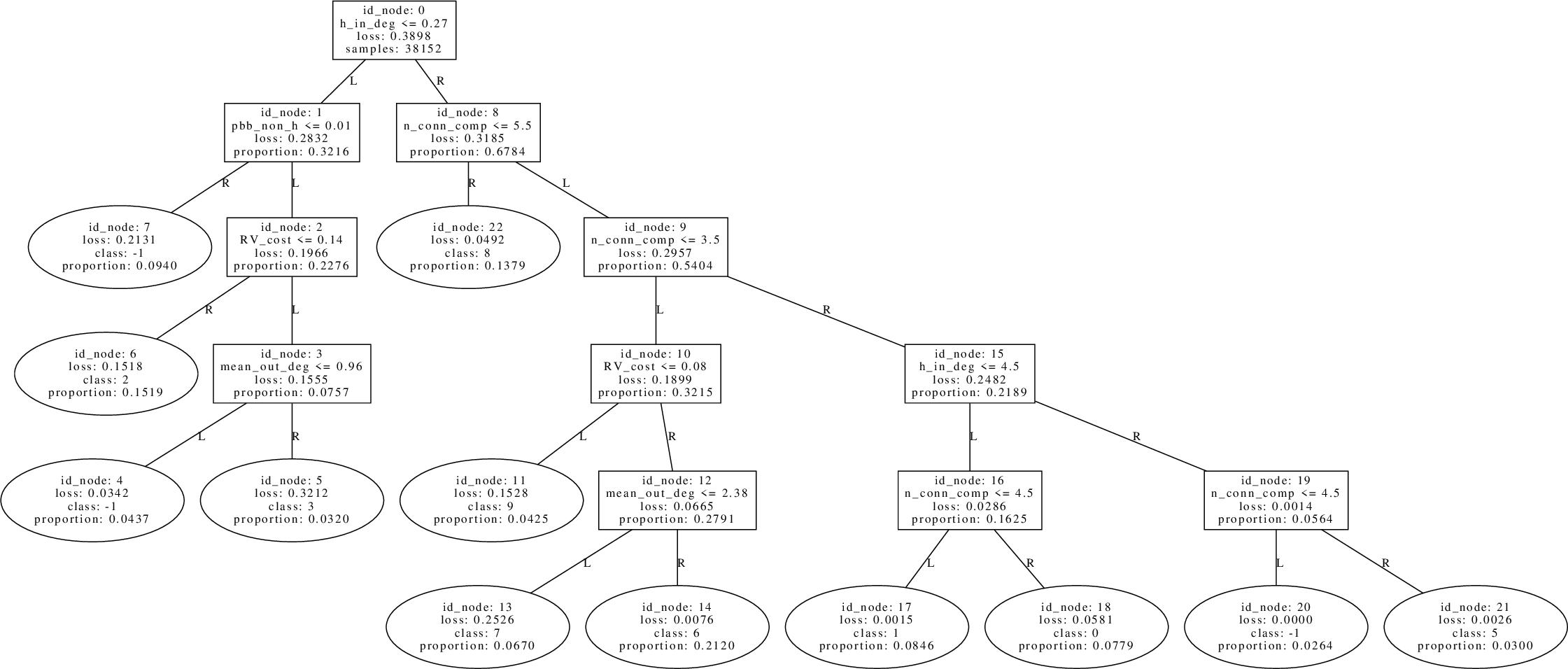

Supplement: S2 Fig — Statistics in the tree are computed on the training set, while average accuracy is computed in the test set. See S3 Appendix of S1 File for the sizes of the datasets used, and Paragraph Description of clusters by classification trees for the general procedure of tree construction. See the first paragraph from Section Welfare optima for an explanation of the variables displayed in tree nodes. (TIF) [file pone.0272733.s002.tif]

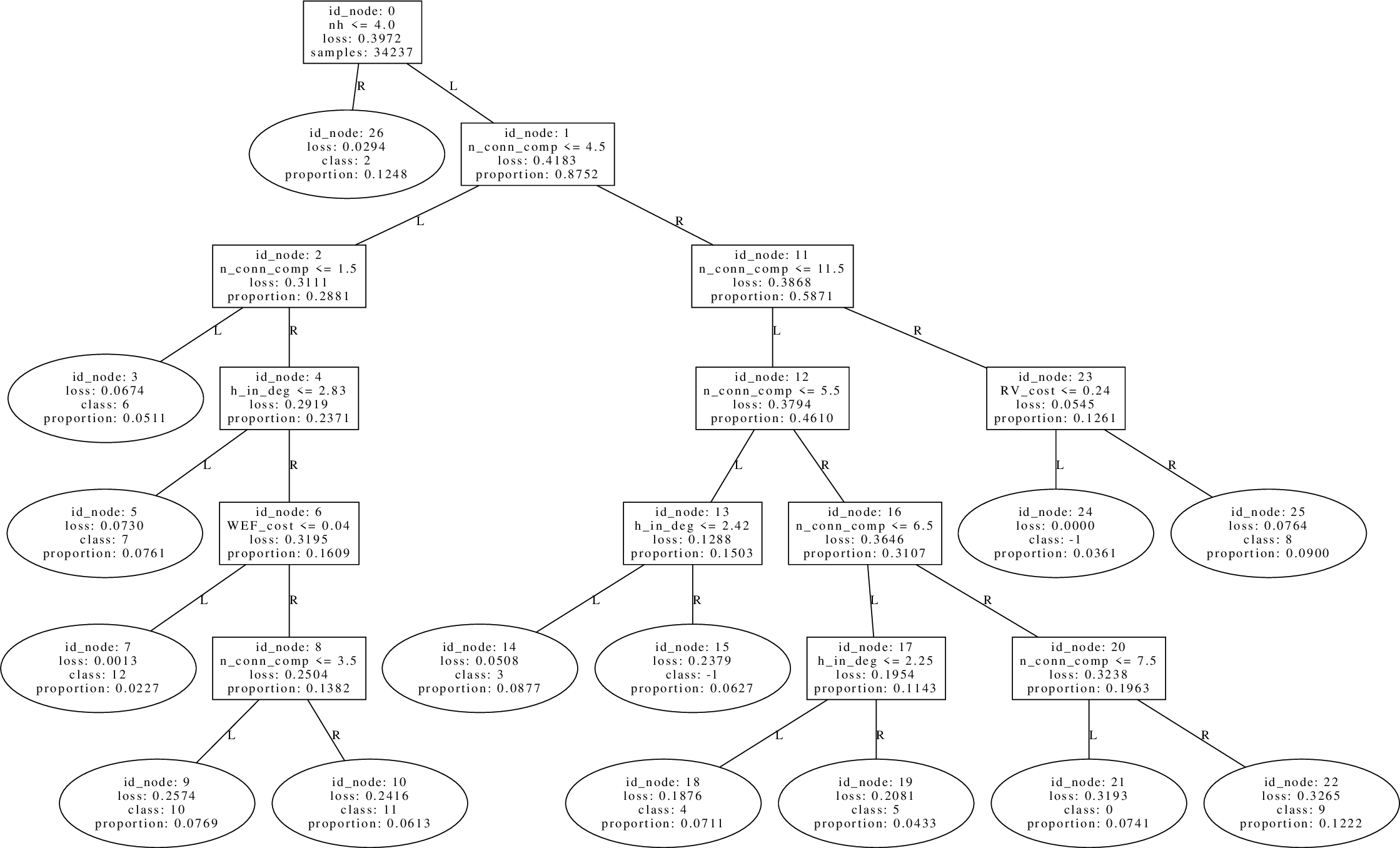

Supplement: S3 Fig — Statistics in the tree are computed on the training set, while average accuracy is computed in the test set. See S3 Appendix of S1 File for the sizes of the datasets used, and Paragraph Description of clusters by classification trees for the general procedure of tree construction. See the first paragraph from Section Welfare optima for an explanation of the variables displayed in tree nodes. (TIF) [file pone.0272733.s003.tif]

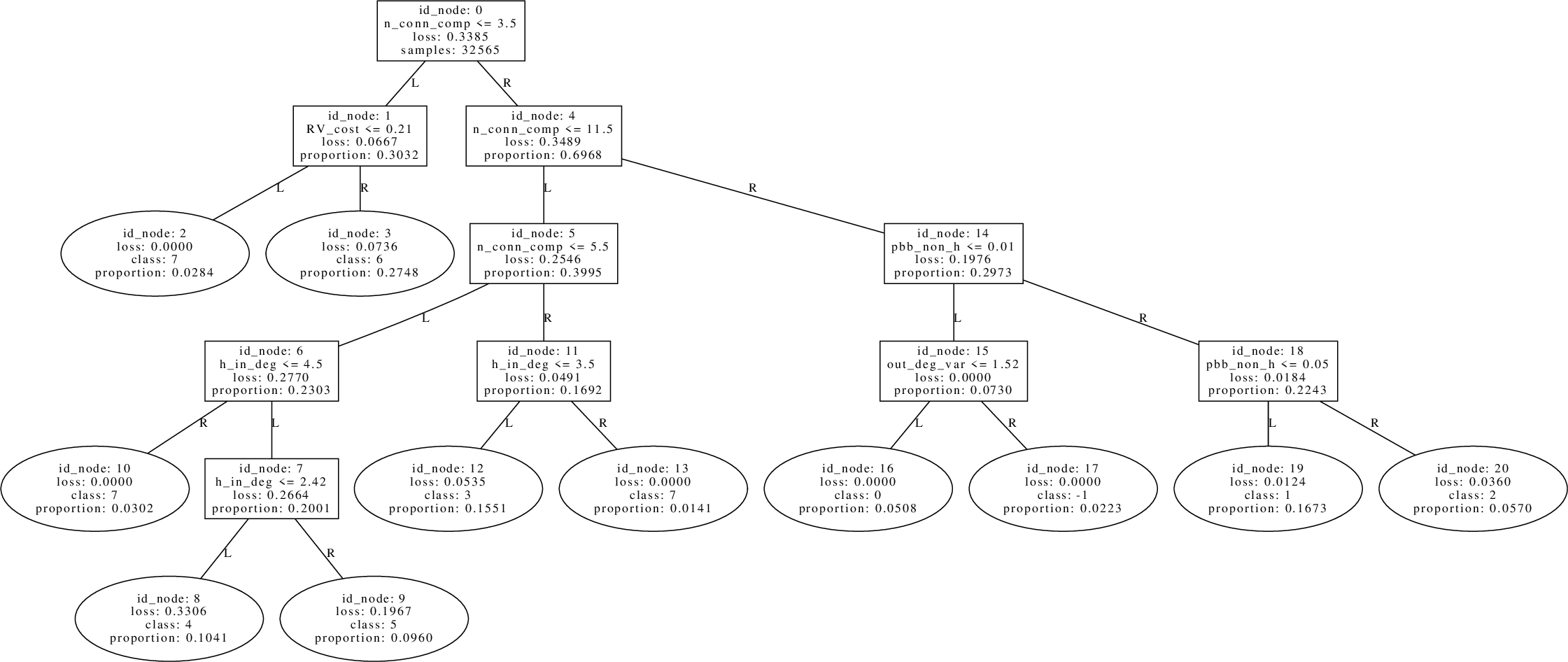

Supplement: S4 Fig — Statistics in the tree are computed on the training set, while average accuracy is computed in the test set. See S3 Appendix of S1 File for the sizes of the datasets used, and Paragraph Description of clusters by classification trees for the general procedure of tree construction. See the first paragraph from Section Welfare optima for an explanation of the variables displayed in tree nodes. (TIF) [file pone.0272733.s004.tif]
