# Supplementary material for: Modularity of food-sharing networks minimises the risk for individual and group starvation in hunter-gatherer societies
Source: PLoS One. 2023 May 10;18(5):e0272733. doi: 10.1371/journal.pone.0272733 (PMC10171659; doi:10.1371/journal.pone.0272733)
Supplement: S1 File — (ZIP) [file pone.0272733.s005.zip › Supporting_Information.pdf]

Modularity of food-sharing networks minimises the risk for individual and group starvation in hunter-gatherer societies

Francisco Plana[1*¤] Jorge Pérez[2] Andrés Abeliuk[1,3]

1 Department of Computer Science, Universidad de Chile, Santiago, Chile.
2 Millennium Institute Foundational Research on Data, Santiago, Chile.
3 National Center for Artificial Intelligence (CENIA), Santiago, Chile.

¤Current Address: Departamento de Ciencias de la Computación, Facultad de Ciencias Físicas y Matemáticas, Universidad de Chile, Santiago, Chile.
* E-mail: franciscoplana@gmail.com (FP)

Supporting information

S1 Appendix. Estimate of probability of eating. In this section, we obtain an estimate of the probability of eating for every node, according to the model assumptions and food sharing protocol. With this goal in mind, we introduce some basic graph notions. We can establish an equivalence between a digraph $D = (V, E)$ of N vertices and its *adjacency matrix*,

$$\mathcal{M}(D)_{i,j} = \begin{cases} 1, & (i,j) \in E \\ 0, & \text{otherwise} \end{cases}$$

and we can define a finite *walk* w in a digraph as a sequence of edges $\{e_i\}_{i=1}^{n-1}$ for which there is a sequence of vertices $\{v_i\}_{i=1}^{n}$, possibly repeated, such that $e_i = (v_i, v_{i+1}), \forall i = 1, \ldots, n-1$. If w is a walk with vertex sequence $\{v_i\}_{i=1}^{n}$, we say that w is a walk *from* v_1 *to* v_n *of length* $n-1$, or a $(n-1)-$walk. It is not difficult to prove that, coefficient $\mathcal{M}(D)_{i,j}^{n}$ from the $n-$th power of $\mathcal{M}(D)$, is equal to the number of walks of length n from i to j in D. We will use this property in what follows. For completeness, we can say that $\mathcal{M}(D)^0 = I_{N\times N}$, the identity matrix, for every network D.

Now, in order to obtain an expression for $pe(v)$, the probability of eating, at each time step, by agent v, let us assume by now that there is just one hunter h to derive an expression for the probability $pe(v,h)$ of v getting feed from the only hunter h. It turns out that it is simpler to write this probability as a function of the negation of the complement event, which corresponds to v not being reached by a $(F-1)-$walk W of nodes sharing a prey from h, or also to the intersection of events $E_i, i = 0, \ldots, F-1$, where each $E_i = \{v_i \neq v | v_i \text{ is the end of the } i-\text{th sub-walk } W_{h,v_i}^{i}\}$. Thus, probability $pe(v,h)$ may be written as follows,

$$pe(v,h) = ph \cdot \left(1 - \mathbb{P}\left(\bigcap_{i=0}^{F-1} E_i\right)\right) \tag{1}$$

$$= ph \cdot \left(1 - \mathbb{P}(E_0) \cdot \prod_{i=1}^{F-1} \frac{\mathbb{P}(\cap_{k=0}^{i} E_k)}{\mathbb{P}(\cap_{j=0}^{i-1} E_j)}\right) \tag{2}$$

$$= ph \cdot \left(1 - (1 - \mathbb{1}_{\{v=h\}}) \cdot \prod_{i=1}^{F-1} \frac{\sum_{x\in\{1,\ldots,N\}} (\mathcal{M}_{(v)}^{i})_{h,x}}{\sum_{x\in\{1,\ldots,N\}} (\mathcal{M}_{(v)}^{i-1} \cdot \mathcal{M})_{h,x}}\right), \tag{3}$$

where the second equality comes from repeated application of the definition of conditional probability. The third equality comes from the definition of probability as the ratio of favorable outcomes and total cases. For both probabilities we can take the same number of total cases as the number of $i-$walks, which cancel in the division and thus only the favorable cases survive. In the first probability, the favorable cases are the number of $i-$walks from h never getting v, which are estimated by the $i-$th power of matrix $\mathcal{M}_{(v)}$, the adjacency matrix of D where all ingoing and outgoing arcs of v are removed. Favorable cases at denominator is just the number of $i-$walks from h never touching v except maybe in their last step. If there are no $i-$walks from h in D, for consistency we define this ratio of probabilities to be 1. Now, when there are $H \geq 1$ hunters $\{h_i\}_{i=1}^{H}$, the *Inclusion-Exclusion Principle* allows us to generalize the probability of eating $pe(v, h_i)$ from hunter h_i, to the probability of getting food from some hunter $pe(v)$,

$$pe(v) = \sum_{k=1}^{H} (-1)^{k-1} \sum_{I \subseteq \{1,\ldots,H\}, |I|=k} \prod_{i \in I} pe(v, h_i). \tag{4}$$

This expression is valid since we have assumed that generation of food by every hunter correspond to independent events.

S2 Appendix. Structure of simulation and sampling of model variables. The methodology we use to analyze our model is to sample a representative set of values for the model variables, obtain for each combination of model variables the respective set of optima via evolutionary algorithms, and then describe these optima with the pipeline of analysis. We use as a study case a population size of $N = 12$, since it is one of the smallest numbers allowing for exploring combinations of number of groups ng and group sizes gs, where both may hold that $ng, gs \geq 3$ and simultaneously one of them may be strictly greater than 3. These magnitudes are enough to represent the network of food transfers of a typical cluster of households, each formed by 3 to 4 families, and each family usually comprised by an adult couple and their dependent children [21]. It is clear that using larger population sizes may produce a wider diversity of structures that may qualify as optimal networks for the model, and that it is not straightforward to define a similarity notion between classes of optimal networks from distinct population sizes in order to assess the scalability of results obtained for a particular network size. However, the empirical fact that larger camps or hunter-gatherer communities are comprised by larger numbers of clusters rather than by larger clusters [21], is suggestive that there may be some kind of spatial constraints in play, such as food productivity by land unit or land limits to household habitability, that shape larger camps as an aggregation of typical-size-clusters. Exploring these spatial constraints is out of the scope of our work, and our approach is to explore the possible network organization with the typical size of a cluster of households.

Regarding a possible parameterization of a limited number of parameters, we have only fixed the RV function by fixing the values of k and n -the critical time period and the life span-. Since distinct RV curves have a similar behavior in general terms, see Fig 2, we estimate that fixing this function does not impose a hard constraint to the analysis, because it only affects the ranges of ph where distinct regimes are observed. We do not have any *a priori* hypothesis on a potential dependence of model variables, and nor know whether it may simplify the analysis. The previous evolutionary model CURP [75], which models the emergence of food-sharing in function of resource pressure and has two variables similar in functionality to ph and F (*prob-resource* to ph, and *min-energy* to F), does not give evidence that these two variables could be easily simplified into one variable. However, on S6 Appendix there is an approximation for the mean probability

of eating for WEF optima, $\bar{pe(v)} \sim \frac{ph}{N} \cdot (F \cdot nh)$, which we conjecture that may serve to establish some invariant on WEF networks with different sizes and similar values of this mean probability. Based on this expression, we have chosen the approach of separately sampling, on the one hand, ph, and on the other hand, F and nh. The intuition to sample the first variable will come from examining the RV function which we will work with. In the case of the last two variables, it comes from the fact that the product $(F \cdot nh)$ represents the maximum of times some food is ever shared in the network. Hence, whether this amount is enough to feed the whole network, that is, whether $(F \cdot nh) < N$, is an important variable for network organization of WEF optima.

The intuition to sample the ph values is the following. The reduction of variability function, which we work with has a maximum value at approximately $pe^{\star} = 0.0899$. Since non-hunter nodes usually eat with a probability smaller than ph, the simpler way of minimizing RV for values of ph smaller than $pe^{\star}$, is that non-hunters just do not receive food and are isolated nodes. This trend is progressively reversed if $ph > pe^{\star}$, since there is a cost in increased RV mean by leaving behind non-hunters, which is accentuated for greater values of ph where RV reaches its minimum values. Following this intuition, we have chosen the values $\{0.02, 0.08, 0.15, 0.3, 0.6\}$ of ph by choosing two smaller, two greater than $pe^{\star}$, and one at the RV flat region. Now, for the other two variables, we have chosen a set of 10 value pairs (F, nh), which is balanced with respect to condition $F \cdot nh < N = 12$. Since there are more combinatorial network-walks possibilities when $F > 2$ and this makes these cases more interesting, the specific value of F constrains nh to only certain possible values in the case $F \cdot nh < N$. This constraint for nh is not present if $F \cdot nh \geq N$. On the other hand, we conjecture that for general networks of size N, the greatest variations in the probability of eating are produced by the range $F \in \{2, \ldots, N/2\}$, and that larger values of F produce smaller variations. Guided by these assumptions, we have sampled the following 10 pairs of variables (F, nh).

$$\{(12, 1), (4, 3), (3, 5), (3, 7), (4, 9), (4, 1), (4, 2), (5, 2), (3, 3), (2, 5)\}$$

We have taken each possible combination of the 50 (ph, F, nh) values as initial condition for the evolutionary network minimization of criteria combination.

S3 Appendix. Sizes of tables of networks and features. The single optima datasets, for RV and WEF, both were appropriately processed by tSNE clustering method, by gathering the data of all conditions of model variables in a single table. The table sizes are the following. The number of features are explained in the section describing them.

Table 1. Dimensions of single criteria optimal networks datasets.

Optima type	Number of networks	Number of features
RV	6047	10
WEF	18686	12

In the case of Pareto optimal networks, however, the set of networks is too diverse to be properly handled in a single table. For this reason, the networks data was split according to the value of ph and the condition whether $F \cdot nh \leq N$. See the paragraph *Summary of distinct types of WEF optima* from Section *Welfare optima*, for the result justifying the use of this last condition. Having said that, below are the sizes of tables used to cluster the datasets of Pareto optimal networks. All these tables have the same feature set used in the clustering of RV data. All datasets and code used to generate them may be consulted in the repository provided for this purpose [72].

Table 2. Dimensions of multicriteria optimal networks datasets.

ph **value**	$F \cdot nh \leq N$ **condition**	**Number of networks**
0.02	True	54503
0.02	False	13642
0.08	True	46522
0.08	False	14703
0.15	True	48910
0.15	False	1852
0.3	True	11082
0.3	False	611
0.6	True	3186
0.6	False	109

S4 Appendix. Domain of optimization model and evolutionary algorithm implementation details. As an example of the situation of parts of a network that do not contribute to network cost, consider the networks of size 6 in Fig with node hunters $\{0, 3\}$. Networks D_1 and D_2 are clearly non-isomorphic. But if we work with $F = 3$, the probabilities of eating only depend on the walks of length 2 starting from the hunters, which in D_1 are $\{(0, 1), (1, 2)\}$, $\{(3, 2), (2, 4)\}$, while in D_2 these are $\{(3, 2), (2, 1)\}$, and $\{(0, 1), (1, 4)\}$. These walks produce the same probabilities of eating for every node under the map $\pi : V_1 \to V_2$, $\pi(0) = 3$, $\pi(3) = 0$, $\pi(1) = 2$, $\pi(2) = 1$, $\pi(4) = 4$ and $\pi(5) = 5$, and in this way, the two networks have exactly the same value for the two criteria. Since the probabilities of eating depend on the reachability from hunters by $(F - 1)-$walks, there will be a huge number of non-isomorphic networks that have the same objective value, due to subgraphs that are not reachable via $(F - 1)-$walks starting from hunter nodes, as we have seen in this example. We remedy this by focusing on those regions of a network which indeed influence the probabilities of eating. Specifically, we will work with the following notion of isomorphism.

S1 Fig. Two non-isomorphic networks with the same cost for $F = 3$. Networks D_1 (left) and D_2 (right). Hunters $\{0, 3\}$ are filled in yellow.

Definition 0.1 (Isomorphism with hunters)**.** Let D, G two digraphs of N nodes, and two respective sets of hunters $H(D) \subseteq V(D)$, and $H(G) \subseteq V(G)$, of equal size, $|H(G)| = |H(D)|$. Let $F \in \mathbb{N}$ with $F \geq 2$. We say that D and G are *$F-$isomorphic with hunters*, if there is a bijection $\pi : V(D) \to V(G)$, satisfying the following:

- The set of hunters is an invariant under the bijection, that is, $\pi(H(D)) = H(G)$.
- For every $u \in V(D)$ at distance at most $(F - 2)$ to some $h \in H(D)$, it holds that $(u, v) \in E(D)$ if and only if $(\pi(u), \pi(v)) \in E(G)$.

It is not difficult to see that the isomorphism with hunters is an equivalence relation in the set of directed graphs (ie, a binary reflexive, symmetric and transitive relation), and hence the set of digraphs can be partitioned into equivalence classes of isomorphic networks having the same costs, even though they may have distinct number of arcs, as seen in the last example. Given these elements, we claim that the *domain set for solution points* of our model corresponds to the set of representative digraphs of the isomorphism equivalence classes. This definition aims to discard portions of the digraph that do not contribute to the objective value.

We next review some implementation decisions we have taken in order to execute the evolutionary algorithms more efficiently, including the mutation procedure which leverages the definition of the domain of the problem we just reviewed. The individuals

in the evolutionary algorithm, or in other words, the networks, are equipped with a data-structure that stores one vector for each hunter, that keeps the updated distance of each network node from the respective hunter. This data structure enables for implementing feasible mutations as those random arc additions or deletions that produce networks belonging to the set of feasible solutions, which reduces the exploration space in comparison to performing arbitrary random arc changes. Distance updates are made by running BFS-like (*breadth-first-search*) algorithms from the respective hunter when a relevant arc addition or deletion has been produced. The reader interested in consulting the specifics of the evolutionary algorithms used may review the code [72] written to perform the simulations.

Other important optimizations are those relative to the data-structure that stores the optima candidates of the optimization, which we will refer to as the *archive.* At each generation, when a population is reviewed to update the archive, the population is filtered, and only those individuals passing the filter are reviewed for possible addition to the archive. In the case of single objective optimization, the filter is being a local optima for the respective function. In the multi objective case, the offspring population is processed by NonDominatedSort, an efficient algorithm that performs a ranking according to Pareto domination [84], thus only the set of individuals that most likely belong to the Pareto optimal set are tested to be added to the archive. Now, it is straightforward to maintain in the archive the updated set of non-dominated individuals processed. However, there is no direct way to determine whether an individual belongs to the Pareto optimal set. Therefore, we have implemented an adaptive heuristic to determine when to stop the generation process such that the archive likely stores a high proportion of Pareto optimal networks. This heuristic is rooted on our empirical observation on exhaustive optima search on networks of small size ($N = 5$), that the number of dominated networks that are erased from the archive at each iteration, becomes negligible with respect to the size of the archive, when the latter converges to a high proportion of Pareto optimal networks. Thus, our heuristic computes the ratio of the standard deviation of erased networks in the last and antepenultimate iteration windows of size $w = 10$, and the current size of the archive. The evolution is stopped if these 2 ratios are smaller than 0.005. On the other hand, single-objective minimization used 2000 generations.

S5 Appendix. Choice of tSNE hyperparameters and heuristics to set OPTICS hyperparameters. The choice of parameters is critical to accomplish a good visualization with tSNE. Since we work with relatively large datasets, we have used the following recommendations [85]: perplexity as 1% of the dataset size n -or the number of networks-, PCA initialization, high learning rate of n/EE, where EE is the early exaggeration, which is chosen usually as 4 or a small number to speed the convergence to solution [86]. We found that this parameter instantiation yielded robust results for distinct random seeds. We used the sci-kit learn implementation of tSNE [87] together with the extension intelex (https://intel.github.io/scikit-learn-intelex/) to accelerate computations of this library.

Now, a cluster in OPTICS is the set of all *density-reachable* objects, or objects connected by regions of similar density, from an arbitrary *core object*, which is a point with at least $MinPts$ points in a neighborhood of radius ϵ named as the *reachability-distance.* OPTICS creates an ordering of the database, which enables producing cluster memberships in linear time for any given $\epsilon \geq 0$. This parameter determines that objects with a reachability-distance greater than ϵ, to their closest density-reachable core object, are considered to be noise. We have chosen $MinPts$ to be the 3% of the dataset size, a relatively large magnitude which reduces the number of core points, and hence, the number of clusters. In order to choose ϵ, we have

implemented the following iterative heuristic, which looks for an epsilon producing a minimun of intra-cluster dispersion in features, while keeping low: the size of noise, and its heterogeneity. At each step, pick the greatest reachability distance in the dataset, compute ϵ as the average of this distance and the greatest distance of last iteration, and obtain respective clustering with this ϵ. Compute the average, over clustering classes, of the sum of intra-class feature standard deviation, for 3 cases: all the labels ($\bar{\sigma}_a$), all the labels except noise ($\bar{\sigma}_{a'}$) and only noise ($\bar{\sigma}_n$). Store the current ϵ if minimizes $\bar{\sigma}_a$ and $\bar{\sigma}_n < rst \cdot \bar{\sigma}_{a'}$, where rst stands for *ratio of standard deviation*. Remove object with the greatest distance and go to next iteration, up to removing a proportion mnp -for *maximum noise permitted*- of the original dataset.

Now, the values of mnp, rst are usually around 0.02 and 1.5. These are the values used for single optima datasets. However, in multicriteria optima datasets, due to greater diversity, these values were checked and eventually reassigned by inspecting that the visual aspect of OPTICS clusters of tSNE map, was not too raw, with a few overly large clusters. This is usually accomplished with a value of $\bar{\sigma}_a$ approximately between 25 and 35. The parameters used for multicriteria optima are in the table below. All code implementing this heuristics may be consulted in the repository provided for this purpose [72].

Table 3. Parameters for OPTICS heuristics on multicriteria optima.

ph **value**	$F \cdot nh \leq N$ **condition**	mnp **parameter**	rst **parameter**
0.02	True	0.02	4
0.02	False	0.02	3
0.08	True	0.02	3
0.08	False	0.03	4.5
0.15	True	0.03	3
0.15	False	0.07	4.5
0.3	True	0.02	4
0.3	False	0.11	3
0.6	True	0.03	7.5

S6 Appendix. Analytical argument for the inclusion of additional features in the construction of decision trees. The quantity $F \cdot nh$, which intuitively represents the maximum of times some food is ever shared in the network, approximately determines the mean of the probability of eating for WEF optima, as we review next. If we start from (4) to write the mean probability of eating for v non-hunters, and since $pe(v, h_i) \in (0, 1), \forall i$, we can take a first order approximation by neglecting products of $pe(v, h_i)$ for $k > 1$, leading to

$$pe(\bar{v}_{\text{non-h}}) \sim \sum_{i=1}^{H} pe(v, h_i) = nh \cdot pe(v, h) \sim nh \cdot \frac{(F - 1) \cdot ph}{(N - nh)}, \tag{5}$$

where the last approximation comes from the equidistribution, on WEF optima, of $(F - 1)$ events of food sharing with probability ph, over a total of $(N - nh)$ non-hunters. Thus, an expression for the mean probability of eating may be written by splitting the mean into hunters and non-hunters, and replacing by (5)

$$\overline{pe}(v) = \frac{nh \cdot ph}{N} + \frac{(N - nh)}{N} \cdot pe(\bar{v}_{\text{non-h}}) \tag{6}$$

$$\sim \frac{ph}{N} \cdot (F \cdot nh). \tag{7}$$

Equation (6) justify our decision to add some model variables to the feature set employed to train the classification trees.

S7 Appendix. Implementation details of decision trees. We implemented the model of decision trees we have used in Python, starting from an open-source implementation of model-trees available on the following site.

`https://github.com/cerlymarco/linear-tree`

We measure syntactic stability of a tree following the approach by Dunne et al (2002) [88] where stability is defined as the average of some similarity measure over the set of distinct pairs of trees belonging to a set of M tree instantiations. The DT similarity measure employed is minus the tree-edit distance of the pair of trees, computed by the APTED algorithm [89] and implemented by an open-source project that may be found in the site below.

`https://pypi.org/project/apted/`

In order to avoid a large number of similar trees with similar values of accuracy and stability, we have grouped those trees that have the same structure and split variables into the same tree class, on which the average of stability and accuracy is computed. We have used $M = 15$ tree instantiations to compute the accuracy and stability pair for each set of DT hyperparameters, where each instantiation comes from a distinct seed of bootstrap sampling. The set of $HP = 1000$ DT hyperparameters is obtained via a random uniform search [90] over the entire set of hyperparameters, which is described in the following table. These ranges were chosen by empirically checking whether they give rise to high accuracy values, usually superior to 0.7. Floating points for number of samples in this table are interpreted as proportions of the dataset. All code implementing these classification trees may be consulted in the repository provided for this purpose [72].

Table 4. Ranges of decision tree hyperparameters.

Hyperparameter name	lower limit	upper limit
minimum of samples to perform a split	$3 * 10^{-5}$	$3 * 10^{-5} + 10^{-3}$
minimum of samples per leaf	$3 * 10^{-5}$	$3 * 10^{-5} + 10^{-3}$
maximum depth	3	8
minimum impurity decrease	0	0.01

Finally, since we work with accuracy and stability sample averages that aim to approximate population parameters, we used the following procedure to determine statistical Pareto-dominance. These sample averages follow approximately a normal distribution, which allows for performing the usual $t-$tests for mean comparison. In order to determine if the pair $\vec{u} = (a_u, s_u)$ Pareto-dominates $\vec{v} = (a_v, s_v)$ (as a maximization), we compute a $2-$sample $1-$tail mean $t-$test for accuracy and stability. If the null hypothesis is rejected, that is, we cannot reject that $a_u < a_v$ or $s_u < s_v$, we know that $\vec{u}$ does not Pareto-dominate $\vec{v}$. Otherwise, compute a $2-$sample $2-$tail mean $t-$test for accuracy and stability. If the null is rejected, that is, we cannot reject that $a_u \neq a_v$ or $s_u \neq s_v$, we conclude that $\vec{u}$ Pareto-dominates $\vec{v}$.

S8 Appendix. Decision trees for Pareto optimal networks. Networks on Fig 11 correspond to the most central networks on the following leaves. Fig 11 (a) from leaf 22 on tree at S2 Fig. Fig 11 (b) from leaf 14 on tree at S3 Fig. Fig 11 (c) from leaf 19 on tree at S4 Fig. The interested reader in examining these or other trees describing sets of optimal networks, may review the documentation for running the code from the repository provided for this purpose [72].

S2 Fig. An efficient tree (accuracy = 0.747) to discriminate the clustering labels of PF optima on $ph = 0.02$**.** Statistics in the tree are computed on the training set, while average accuracy is computed in the test set. See S3 Appendix for the sizes of the datasets used, and Paragraph *Description of clusters by classification trees* for the general procedure of tree construction. See the first paragraph from Section *Welfare optima* for an explanation of the variables displayed in tree nodes.

S3 Fig. An efficient tree (accuracy = 0.734) to discriminate the clustering labels of PF optima on $ph = 0.15$**.** Statistics in the tree are computed on the training set, while average accuracy is computed in the test set. See S3 Appendix for the sizes of the datasets used, and Paragraph *Description of clusters by classification trees* for the general procedure of tree construction. See the first paragraph from Section *Welfare optima* for an explanation of the variables displayed in tree nodes.

S4 Fig. An efficient tree (accuracy = 0.845) to discriminate the clustering labels of PF optima on $ph = 0.08$**.** Statistics in the tree are computed on the training set, while average accuracy is computed in the test set. See S3 Appendix for the sizes of the datasets used, and Paragraph *Description of clusters by classification trees* for the general procedure of tree construction. See the first paragraph from Section *Welfare optima* for an explanation of the variables displayed in tree nodes.

References

1. Newman ME, Park J. Why social networks are different from other types of networks. Physical review E. 2003;68(3):036122.

2. Keeley LH. Hunter-gatherer economic complexity and "population pressure": A cross-cultural analysis. Journal of anthropological archaeology. 1988;7(4):373–411.

3. Gurven M, Jaeggi AV. Food sharing. Emerging trends in the social and behavioral sciences: An interdisciplinary, searchable, and linkable resource. 2015; p. 1–12.

4. Jaeggi AV, Gurven M. Natural cooperators: food sharing in humans and other primates. Evolutionary Anthropology: Issues, News, and Reviews. 2013;22(4):186–195.

5. Kaplan H, Hill K, Lancaster J, Hurtado AM. A theory of human life history evolution: Diet, intelligence, and longevity. Evolutionary Anthropology: Issues, News, and Reviews: Issues, News, and Reviews. 2000;9(4):156–185.

6. Hamilton MJ. Collective Computation, Information Flow, and the Emergence of Hunter-Gatherer Small-Worlds. Journal of Social Computing. 2022;3(1):18–37.

7. Migliano AB, Vinicius L. The origins of human cumulative culture: from the foraging niche to collective intelligence. Philosophical Transactions of the Royal Society B. 2022;377(1843):20200317.

8. Kramer KL, Ellison PT. Pooled energy budgets: Resituating human energy-allocation trade-offs. Evolutionary Anthropology: Issues, News, and Reviews. 2010;19(4):136–147.

9. Hill K, Hurtado AM. Cooperative breeding in South American hunter–gatherers. Proceedings of the Royal Society B: Biological Sciences. 2009;276(1674):3863–3870.

10. Patton JQ. Meat sharing for coalitional support. Evolution and human behavior. 2005;26(2):137–157.

11. Kent S. Sharing in an egalitarian Kalahari community. Man. 1993; p. 479–514.

12. Hooper PL, Gurven M, Winking J, Kaplan HS. Inclusive fitness and differential productivity across the life course determine intergenerational transfers in a small-scale human society. Proceedings of the Royal Society B: Biological Sciences. 2015;282(1803):20142808.

13. Ready E, Power EA. Why wage earners hunt: food sharing, social structure, and influence in an Arctic mixed economy. Current Anthropology. 2018;59(1):74–97.

14. Ringen EJ, Duda P, Jaeggi AV. The evolution of daily food sharing: A Bayesian phylogenetic analysis. Evolution and Human Behavior. 2019;40(4):375–384.

15. Bailey RC. The behavioral ecology of Efe Pygmy men in the Ituri Forest, Zaire. 86. University of Michigan Museum; 1991.

16. Smith EA, Bird RLB. Turtle hunting and tombstone opening: Public generosity as costly signaling. Evolution and human behavior. 2000;21(4):245–261.

17. Hawkes K, Bliege Bird R. Showing off, handicap signaling, and the evolution of men's work. Evolutionary Anthropology: Issues, News, and Reviews: Issues, News, and Reviews. 2002;11(2):58–67.

18. Apicella CL, Marlowe FW, Fowler JH, Christakis NA. Social networks and cooperation in hunter-gatherers. Nature. 2012;481(7382):497–501.

19. von Rueden CR, Redhead D, O'Gorman R, Kaplan H, Gurven M. The dynamics of men's cooperation and social status in a small-scale society. Proceedings of the Royal Society B. 2019;286(1908):20191367.

20. Koster J. Interhousehold meat sharing among Mayangna and Miskito horticulturalists in Nicaragua. Human Nature. 2011;22(4):394–415.

21. Dyble M, Thompson J, Smith D, Salali GD, Chaudhary N, Page AE, et al. Networks of food sharing reveal the functional significance of multilevel sociality in two hunter-gatherer groups. Current Biology. 2016;26(15):2017–2021.

22. Migliano AB, Page AE, Gómez-Gardeñes J, Salali GD, Viguier S, Dyble M, et al. Characterization of hunter-gatherer networks and implications for cumulative culture. Nature Human Behaviour. 2017;1(2):1–6.

23. Derex M, Boyd R. Partial connectivity increases cultural accumulation within groups. Proceedings of the National Academy of Sciences. 2016;113(11):2982–2987.

24. Derex M, Godelle B, Raymond M. Social learners require process information to outperform individual learners. Evolution: International Journal of Organic Evolution. 2013;67(3):688–697.

25. Camerlenghi E, McQueen A, Delhey K, Cook CN, Kingma SA, Farine DR, et al. Cooperative breeding and the emergence of multilevel societies in birds. Ecology letters. 2022;.

26. Nolin DA. Food-sharing networks in Lamalera, Indonesia. Human Nature. 2010;21(3):243–268.

27. Kasper C, Mulder MB. Who helps and why? Cooperative networks in Mpimbwe. Current Anthropology. 2015;56(5):701–732.

28. Kaplan HS, Schniter E, Smith VL, Wilson BJ. Risk and the evolution of human exchange. Proceedings of the Royal Society B: Biological Sciences. 2012;279(1740):2930–2935.

29. Jaeggi AV, Gurven M. Reciprocity explains food sharing in humans and other primates independent of kin selection and tolerated scrounging: a phylogenetic meta-analysis. Proceedings of the Royal Society B: Biological Sciences. 2013;280(1768):20131615.

30. Hames R, McCabe C. Meal sharing among the Ye'kwana. Human Nature. 2007;18(1):1–21.

31. Allen-Arave W, Gurven M, Hill K. Reciprocal altruism, rather than kin selection, maintains nepotistic food transfers on an Ache reservation. Evolution and Human Behavior. 2008;29(5):305–318.

32. Smith D, Dyble M, Major K, Page AE, Chaudhary N, Salali GD, et al. A friend in need is a friend indeed: Need-based sharing, rather than cooperative assortment, predicts experimental resource transfers among Agta hunter-gatherers. Evolution and human behavior. 2019;40(1):82–89.

33. Von Rueden C, Gurven M, Kaplan H. Why do men seek status? Fitness payoffs to dominance and prestige. Proceedings of the Royal Society B: Biological Sciences. 2011;278(1715):2223–2232.

34. Gurven M, Hill K. Why do men hunt? A reevaluation of "man the hunter" and the sexual division of labor. Current Anthropology. 2009;50(1):51–74.

35. Nolin DA. Food-sharing networks in Lamalera, Indonesia: status, sharing, and signaling. Evolution and Human Behavior. 2012;33(4):334–345.

36. Gurven M, Hill K, Kaplan H, Hurtado A, Lyles R. Food transfers among Hiwi foragers of Venezuela: tests of reciprocity. Human Ecology. 2000;28(2):171–218.

37. Stevens JR, Cushman FA. Cognitive constraints on reciprocity and tolerated scrounging. Behavioral and Brain Sciences. 2004;27(4):569–570.

38. Kaplan H, Hill K. Food sharing among ache foragers: Tests of explanatory hypotheses. Current anthropology. 1985;26(2):223–246.

39. Bergstrom TC. The algebra of assortative encounters and the evolution of cooperation. International Game Theory Review. 2003;5(03):211–228.

40. Bird RB, Bird DW, Smith EA, Kushnick GC. Risk and reciprocity in Meriam food sharing. Evolution and Human Behavior. 2002;23(4):297–321.

41. Gurven M. Reciprocal altruism and food sharing decisions among Hiwi and Ache hunter–gatherers. Behavioral Ecology and Sociobiology. 2004;56(4):366–380.

42. Fuller S. Positivism, history of. In: Smelser NJ, Baltes PB, editors. International encyclopedia of the social and behavioral sciences. Amsterdam: Elsevier; 2001. p. 11821–11827.

43. Leonetti DL, Chabot-Hanowell B. The foundation of kinship. Human Nature. 2011;22(1):16–40.

44. Kaplan HS, Hooper PL, Gurven M. The evolutionary and ecological roots of human social organization. Philosophical Transactions of the Royal Society B: Biological Sciences. 2009;364(1533):3289–3299.

45. McFall JP. Rational, normative, descriptive, prescriptive, or choice behavior? The search for integrative metatheory of decision making. Behavioral Development Bulletin. 2015;20(1):45.

46. Gurven M. Reciprocal altruism and food sharing decisions among Hiwi and Ache hunter–gatherers. Behavioral Ecology and Sociobiology. 2004;56(4):366–380.

47. Hawkes K. Showing off: tests of an hypothesis about men's foraging goals. Ethology and sociobiology. 1991;12(1):29–54.

48. Ahedo V, Caro J, Bortolini E, Zurro D, Madella M, Galán JM. Quantifying the relationship between food sharing practices and socio-ecological variables in small-scale societies: A cross-cultural multi-methodological approach. PloS one. 2019;14(5):e0216302.

49. Chiang YS. Good samaritans in networks: An experiment on how networks influence egalitarian sharing and the evolution of inequality. PloS one. 2015;10(6):e0128777.

50. Jaeggi AV, Burkart JM, Van Schaik CP. On the psychology of cooperation in humans and other primates: combining the natural history and experimental evidence of prosociality. Philosophical Transactions of the Royal Society B: Biological Sciences. 2010;365(1553):2723–2735.

51. Hrdy SB. Evolutionary context of human development: The cooperative breeding model. Family relationships: An evolutionary perspective. 2007; p. 39–68.

52. Kaplan H, Gurven M, Hill K, Hurtado AM. The natural history of human food sharing and cooperation: a review and a new multi-individual approach to the negotiation of norms. Moral sentiments and material interests: The foundations of cooperation in economic life. 2005;6:75–113.

53. Fu J, Koutras M. Distribution theory of runs: a Markov chain approach. Journal of the American Statistical Association. 1994;89(427):1050–1058.

54. Rousseeuw PJ. Tutorial to robust statistics. Journal of chemometrics. 1991;5(1):1–20.

55. Coello CAC, Lamont GB, Van Veldhuizen DA, et al. Evolutionary algorithms for solving multi-objective problems. vol. 5. Springer; 2007.

56. Eiben AE, Smith JE. Introduction to evolutionary computing. Springer; 2015.

57. Fortin FA, Parizeau M. Revisiting the NSGA-II crowding-distance computation. In: Proceedings of the 15th annual conference on Genetic and evolutionary computation; 2013. p. 623–630.

58. Deb K, Pratap A, Agarwal S, Meyarivan T. A fast and elitist multiobjective genetic algorithm: NSGA-II. IEEE transactions on evolutionary computation. 2002;6(2):182–197.

59. Fortin FA, De Rainville FM, Gardner MA, Parizeau M, Gagné C. DEAP: Evolutionary Algorithms Made Easy. Journal of Machine Learning Research. 2012;13:2171–2175.

60. Peixoto TP. The graph-tool python library. figshare. 2014;doi:10.6084/m9.figshare.1164194.

61. Raymaekers J, Rousseeuw P. A generalized spatial sign covariance matrix. Journal of Multivariate Analysis. 2019;171:94–111.

62. Watts DJ, Strogatz SH. Collective dynamics of 'small-world'networks. nature. 1998;393(6684):440–442.

63. Zamora-López G, Zlatić V, Zhou C, Štefančić H, Kurths J. Reciprocity of networks with degree correlations and arbitrary degree sequences. Physical Review E. 2008;77(1):016106.

64. Wasserman S, Faust K. Social network analysis: Methods and applications. vol. 8. Cambridge university press; 1994.

65. Newman ME. Modularity and community structure in networks. Proceedings of the national academy of sciences. 2006;103(23):8577–8582.

66. Peixoto TP. Efficient Monte Carlo and greedy heuristic for the inference of stochastic block models. Physical Review E. 2014;89(1):012804.

67. Van der Maaten L, Hinton G. Visualizing data using t-SNE. Journal of machine learning research. 2008;9(11).

68. Ankerst M, Breunig MM, Kriegel HP, Sander J. OPTICS: Ordering points to identify the clustering structure. ACM Sigmod record. 1999;28(2):49–60.

69. Loh WY. Improving the precision of classification trees. The Annals of Applied Statistics. 2009; p. 1710–1737.

70. Efron B, Tibshirani R. Improvements on cross-validation: the 632+ bootstrap method. Journal of the American Statistical Association. 1997;92(438):548–560.

71. Guyon I, Bennett K, Cawley G, Escalante HJ, Escalera S, Ho TK, et al. Design of the 2015 ChaLearn AutoML challenge. In: 2015 International Joint Conference on Neural Networks (IJCNN); 2015. p. 1–8.

72. Plana F, Pérez J. Food-sharing data and code. Figshare Digital Repository. 2022;doi:10.6084/m9.figshare.19203926.

73. Briz i Godino I, Santos JI, Galán JM, Caro J, Álvarez M, Zurro D. Social Cooperation and Resource Management DynamicsAmong Late Hunter-Fisher-Gatherer Societies in Tierra del Fuego (South America). Journal of Archaeological Method and Theory. 2014;21(2):343–363.

74. Chaudhary N, Salali GD, Thompson J, Rey A, Gerbault P, Stevenson EGJ, et al. Competition for Cooperation: variability, benefits and heritability of relational wealth in hunter-gatherers. Scientific Reports. 2016;6(1):1–7.

75. Pereda M, Zurro D, Santos JI, Briz i Godino I, Álvarez M, Caro J, et al. Emergence and evolution of cooperation under resource pressure. Scientific reports. 2017;7(1):1–10.

76. Kayser K, Armbruster D. Social optima of need-based transfers. Physica A: Statistical Mechanics and its Applications. 2019;536:121011.

77. Mac Carron P, Kaski K, Dunbar R. Calling Dunbar's numbers. Social Networks. 2016;47:151–155.

78. Winterhalder B. Diet choice, risk, and food sharing in a stochastic environment. Journal of anthropological archaeology. 1986;5(4):369–392.

79. Hames R. Reciprocal altruism in Yanomamo food exchange. Adaptation and human behavior: an anthropological perspective. 2000; p. 397–416.

80. Berbesque JC, Wood BM, Crittenden AN, Mabulla A, Marlowe FW. Eat first, share later: Hadza hunter–gatherer men consume more while foraging than in central places. Evolution and Human Behavior. 2016;37(4):281–286.

81. Karlan D, Mobius M, Rosenblat T, Szeidl A. Trust and social collateral. The Quarterly Journal of Economics. 2009;124(3):1307–1361.

82. Wilson DS, Ostrom E, Cox ME. Generalizing the core design principles for the efficacy of groups. Journal of Economic Behavior & Organization. 2013;90:S21–S32.

83. Okasha S. Evolution and the levels of selection. Clarendon Press; 2006.

84. Fortin FA, Grenier S, Parizeau M. Generalizing the improved run-time complexity algorithm for non-dominated sorting. In: Proceedings of the 15th annual conference on Genetic and evolutionary computation; 2013. p. 615–622.

85. Kobak D, Berens P. The art of using t-SNE for single-cell transcriptomics. Nature communications. 2019;10(1):1–14.

86. Belkina AC, Ciccolella CO, Anno R, Halpert R, Spidlen J, Snyder-Cappione JE. Automated optimized parameters for T-distributed stochastic neighbor embedding improve visualization and analysis of large datasets. Nature communications. 2019;10(1):1–12.

87. Pedregosa F, Varoquaux G, Gramfort A, Michel V, Thirion B, Grisel O, et al. Scikit-learn: Machine Learning in Python. Journal of Machine Learning Research. 2011;12:2825–2830.

88. Dunne K, Cunningham P, Azuaje F. Solutions to instability problems with sequential wrapper-based approaches to feature selection. Journal of Machine Learning Research. 2002; p. 1–22.

89. Pawlik M, Augsten N. Tree edit distance: Robust and memory-efficient. Information Systems. 2016;56:157–173.

90. Bergstra J, Bengio Y. Random search for hyper-parameter optimization. Journal of machine learning research. 2012;13(2).
